# Hippo signaling controls cell cycle and restricts cell plasticity in planarians

**DOI:** 10.1371/journal.pbio.2002399

**Published:** 2018-01-22

**Authors:** Nídia de Sousa, Gustavo Rodríguez-Esteban, Jose Ignacio Rojo-Laguna, Emili Saló, Teresa Adell

**Affiliations:** 1 Department of Genetics, Microbiology and Statistics and Institute of Biomedicine, Universitat de Barcelona, Barcelona, Catalunya, Spain; 2 Institut de Biomedicina de la Universitat de Barcelona (IBUB), Universitat de Barcelona, Barcelona, Catalunya, Spain; 3 CNAG-CRG, Centre for Genomic Regulation (CRG), Barcelona Institute of Science and Technology (BIST), Barcelona, Catalunya, Spain; 4 Universitat Pompeu Fabra (UPF), Barcelona, Catalunya, Spain; The Francis Crick Institute, United Kingdom of Great Britain and Northern Ireland

## Abstract

The Hippo pathway plays a key role in regulating cell turnover in adult tissues, and abnormalities in this pathway are consistently associated with human cancers. Hippo was initially implicated in the control of cell proliferation and death, and its inhibition is linked to the expansion of stem cells and progenitors, leading to larger organ size and tumor formation. To understand the mechanism by which Hippo directs cell renewal and promotes stemness, we studied its function in planarians. These stem cell–based organisms are ideal models for the analysis of the complex cellular events underlying tissue renewal in the whole organism. *hippo* RNA interference (RNAi) in planarians decreased apoptotic cell death, induced cell cycle arrest, and could promote the dedifferentiation of postmitotic cells. *hippo* RNAi resulted in extensive undifferentiated areas and overgrowths, with no effect on body size or cell number. We propose an essential role for *hippo* in controlling cell cycle, restricting cell plasticity, and thereby preventing tumoral transformation.

## Author summary

Constant cell renewal is required to maintain healthy organs during adult homeostasis. The highly conserved Hippo signaling pathway is essential for the regulation of basic cell behaviors that underlie tissue renewal, including cell proliferation, cell differentiation, and cell death. The Hippo protein has been implicated in several human cancers, and its inhibition in mouse and *Drosophila* promotes the formation of overgrowths. Nonetheless, its biological function remains poorly understood. To address this issue, we studied the role of Hippo in planarians, flatworms that continuously alter their size depending on nutrient availability, and therefore have a high rate of cellular turnover. This ability is sustained by an abundant population of adult totipotent stem cells. We show that *hippo* inhibition in planarians decreases apoptotic cell death, impairs cell progression through the cell cycle, and causes instability of the differentiated cell fate. These events ultimately lead to the formation of overgrowths consisting of undifferentiated cells. We propose that the main role of Hippo in planarians is not to promote proliferation but to control the cell cycle and maintain a stable differentiated cell fate.

## Introduction

The same developmental processes that drive embryogenesis also regulate the constant cell renewal required throughout the natural life span of the organism. Successful cell renewal relies on multiple events, including proliferation and differentiation of progenitor cell populations and death of unnecessary cells. Failure to correctly coordinate these events can lead to diseases such as cancer. Of the multiple molecular mechanisms involved in the control of cellular renewal, the Hippo signaling pathway has emerged as a key hub. Although first identified as a key regulator of organ size through the control of cell death and proliferation [1–4], growing evidence suggests additional pivotal roles in coordinating stem-cell maintenance, cell differentiation, cell fate decisions, and cell survival [5–10].

At the core of the Hippo pathway is a kinase cascade that phosphorylates the nuclear effector Yorkie (Yki) (YAP/TAZ in vertebrates) and targets it for degradation. When the pathway is inactive, dephosphorylated Yki enters the nucleus to regulate gene expression [11,12]. In most organs and tissues, such as the liver, heart, and skin, loss of Hippo signaling, or elevated activity of Yki/YAP/TAZ, is associated with stem-cell expansion, inhibition of cell differentiation, the appearance of overgrowths, and tumorigenesis [2,13–15]. In line with these observations, YAP/TAZ is hyperactivated in most human cancers [16]. Importantly, in regenerative contexts, YAP/TAZ promotes regeneration of the same organs in which it produces tumors under homeostatic conditions [17,18]. The Hippo signaling pathway therefore appears to exert a general function, promoting stemness or amplifying the population of progenitors, that is beneficial in regenerating tissues but harmful in homeostatic conditions [2, 8,14].

Despite its crucial role in everyday tissue renewal and in the maintenance of healthy organisms, the mechanism by which Hippo signaling promotes stemness remains unclear. Studies performed in several tissue types have extensively demonstrated the positive effect of Hippo inhibition on cell proliferation and the consequent expansion of the resident population of stem cells [7,19–22]. However, recent studies in the liver and intestine, as well as in embryonic stem (ES) and induced pluripotent stem (iPS) cell cultures, have shown that both Hippo down-regulation and YAP/TAZ nuclearization increase the plasticity of differentiated cells, allowing their dedifferentiation towards a stem-cell fate [9,23–25]. The plasticity of cells within the hierarchical organization of a tissue has major implications for regenerative medicine and cancer [26–28].

To better understand the role of the Hippo pathway in driving adult cellular renewal and specifically in promoting cell stemness, we studied its function in planarians. Owing to the presence of a population of pluripotent adult stem cells (called cNeoblasts) [29,30], planarians have the ability to constantly grow and degrow depending on food availability and to regenerate any missing body part within a few days. Several lineage-restricted cycling cells (or lineage-restricted neoblasts) and their postmitotic descendants can be identified in planarians based on the expression of tissue-specific transcription factors [31,32]. In addition to the presence of a stem cell population, the continuous activation of signaling cues that coordinate cell death and cell renewal and direct precise cell fate decisions allow planarians to maintain proportioned and functional organs during growth/degrowth and regeneration. This continuous active regulation of the stem cell and postmitotic cell compartments makes the planarian an ideal in vivo model of the different events underlying homeostatic cell renewal and tissue regeneration. Furthermore, in contrast to most models of regeneration, planarian regeneration is fueled directly by the expansion of an abundant stem cell population, precluding the need for dedifferentiation [33].

Here, we investigate whether down-regulation of Hippo signaling exerts its stemness-promoting effect by increasing the proliferation of resident stem cells or by promoting cell dedifferentiation. We show that inhibition of *Smed-hippo* (referred to hereafter as *hippo*) in planarians reduces apoptotic activity and increases mitotic rates. However, this imbalance between cell death and mitotic activity does not lead to an increase in planarian body size or cell number, possibly because *hippo* RNAi does not increase the number of cycling cells but rather blocks mitotic exit and increases necrotic cell death. *hippo (RNAi)* planarians develop overgrowths and extensive regions composed of undifferentiated cells, accompanied by a general decrease in the number of differentiated cells throughout the body. A detailed study of the epidermal lineage reveals that *hippo* is required to determine the hierarchical transitions necessary for proper epidermal differentiation from epidermal-restricted stem cells to differentiated epidermal cells. Finally, our results indicate that *hippo* is required to maintain the differentiated state in planarian cells, because *hippo* inhibition could promote dedifferentiation of postmitotic cells. Overall, our results indicate that the overgrowths and undifferentiated regions observed after *hippo* inhibition in planarians are not caused by an imbalance between cell death and proliferation but by the inability of cells to reach and maintain the appropriate fate. We propose an essential role for *hippo* in restricting cell plasticity and hence in preventing tumoral transformation.

## Results

### *hippo* controls the number of apoptotic and mitotic cells in planarians

**Fig 1:**
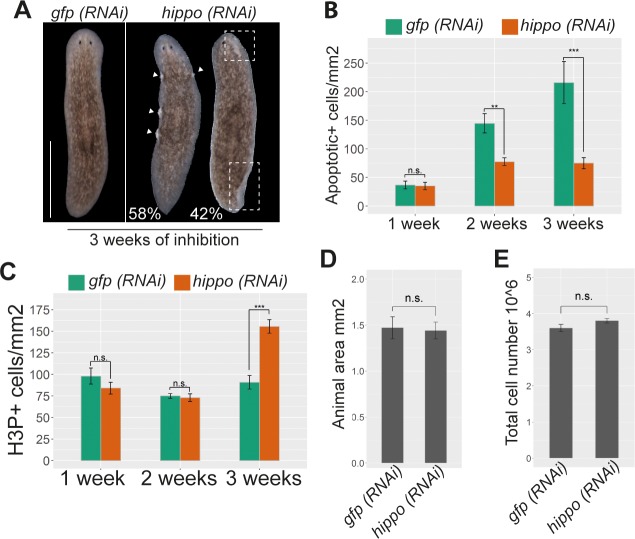
Inhibition of *hippo* in planarians decreases apoptosis and increases mitotic rates but does not affect cell number. (A) Stereomicroscopic image of live *hippo (RNAi)* planarians showing the overgrowths and unpigmented regions in marginal regions of the body (*n* = 30). White arrows indicate overgrowths and white discontinuous boxes indicate unpigmented regions. (B) Quantification of apoptotic cells (TUNEL+) after 1, 2, and 3 weeks of *hippo* RNAi (*n* ≥ 5). (C) Quantification of mitotic cells (H3P+) after 1, 2, and 3 weeks of *hippo* RNAi (*n* ≥ 10). (D) Quantification of body area in planarians subjected to 3 weeks of *hippo* RNAi, as compared with controls (*n* ≥ 8). (E) Graph showing the total cell number in planarians subjected to 3 weeks of *hippo* RNAi, as analyzed by FACS. Bars correspond to the mean of 3 biological replicates. Data used in the generation of this figure can be found in S1 Data. Error bars represent standard deviation. Data were analyzed by Student *t* test. **p < 0.01; ***p < 0.001. FACS, fluorescence-activated cell sorting; *gfp*, *green fluorescent protein*; H3P, phospho-histone-H3-Ser10; n.s., not significant; RNAi, RNA interference.

To study the role of the Hippo pathway in planarians, we conducted a functional analysis of *hippo*, the core element of the pathway, in *Schmidtea mediterranea* [34]. In situ hybridization (ISH) and in silico searches in the single-cell database Planaria SCS [35] indicated that *hippo* is expressed in cells of all types (S1A and S1B Fig). To decipher the possible function of *hippo* during homeostatic cell renewal in planarians, we injected animals with *hippo* double-stranded RNA (dsRNA) for 3 weeks (see Material and methods and S1C Fig). This resulted in a significant decrease in *hippo* mRNA levels beginning during the first week of treatment (S1D Fig). The appearance of unpigmented regions, mainly around the body margin, was observed during the third week of *hippo* inhibition. Over time, these regions became larger or evolved into unpigmented overgrowths (Fig 1A and S1E Fig).

To determine whether the appearance of overgrowths was caused by an imbalance between cell death and cell proliferation, we performed TUNEL and caspase-3 assays and quantified mitotic activity by anti-phospho-Histone 3 immunostaining (H3P). After 2 weeks of inhibition, *hippo (RNAi)* animals exhibited a reduction in cell death compared to controls that became more evident after 3 weeks of inhibition (Fig 1B and S2A and S2B Fig). After 3 weeks of *hippo* RNAi, the number of apoptotic cells was reduced compared to controls, and mitotic activity was increased (Fig 1C and S2C Fig). Nonetheless, measurement of body area revealed no difference between *hippo (RNAi)* planarians and controls (Fig 1A and 1D). Quantification of the total number of cells using fluorescence-activated cell sorting (FACS) or a Neubauer chamber also revealed no differences in cell number between *hippo (RNAi)* and control planarians (Fig 1E and S2D Fig). Our results thus indicate that Hippo promotes apoptotic cell death and controls mitotic activity and that its inhibition leads to the formation of unpigmented regions and overgrowths, without affecting animal size or cell number.

### Hippo is essential for proper cell cycle progression

**Fig 2:**
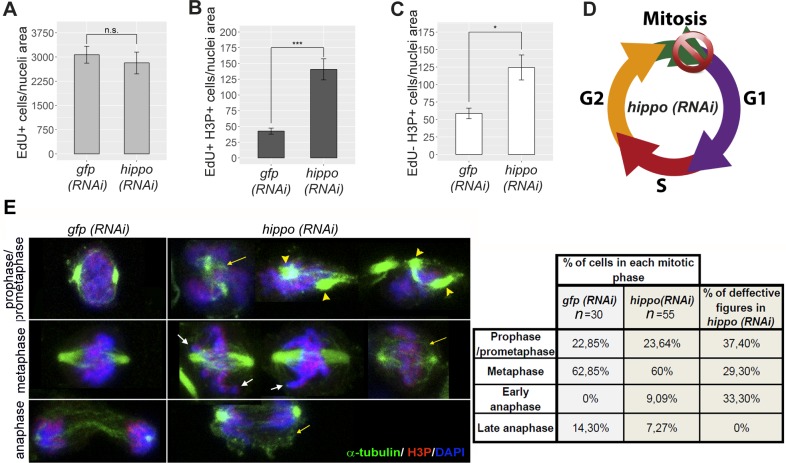
*hippo* plays a pivotal role during the cell cycle. (A) Quantification of EdU+ cells in *hippo (RNAi)* animals and corresponding controls. (B) Quantification of double-positive EdU+/H3P+ cells in *hippo (RNAi)* animals and corresponding controls. (C) Quantification of EdU−/H3P+ cells in *hippo (RNAi)* animals and corresponding controls. Experiments were performed after a 16-h EdU pulse. All graphs correspond to animals subjected to *hippo* RNAi for 3 weeks (*n* ≥ 8). Error bars represent standard deviation. Data were analyzed by Student *t* test. *p < 0.05; ***p < 0.001. (D) Schematic showing the proposed function of *hippo* during the cell cycle in planarians: *hippo* attenuates G2/M progression and ensures successful mitotic exit. (E) α-tubulin arrangement in *hippo (RNAi)* and control cells, as revealed by double immunostaining with anti-α-tubulin and anti-H3P. Nuclei are stained with DAPI. The percentage of each mitotic phase and of defective figures found in *hippo (RNAi)* cells is shown in the table. Yellow arrows indicate disorganized and less dense arrays of spindle microtubules; yellow arrowheads indicate mislocalized poles; white arrows indicate unaligned chromosomes. Data used in the generation of this figure can be found in S1 Data. Scale bar: 10 μm. EdU, 5-ethynyl-2′-deoxyuridine; *gfp*, *green fluorescent protein*; H3P, phospho-histone-H3-Ser10; n.s., not significant; RNAi, RNA interference.

Studies using other animal models have reported that *hippo* dysregulation results in defects in cell cycle progression [36,37]. This effect could provide a plausible explanation for our observation that the decrease in cell death and increase in mitotic activity in *hippo (RNAi)* animals do not lead to changes in cell number. To test this hypothesis, we treated cells with 5-ethynyl-2′-deoxyuridine (EdU) and analyzed the proportion of cells in M and S phase 16 h later in *hippo (RNAi)* animals and corresponding controls (S3A Fig). We found comparable numbers of EdU+ cells in *hippo (RNAi)* animals and controls, indicating that similar numbers of cells enter the cell cycle in both conditions (Fig 2A and S3B Fig). Moreover, double staining with anti-EdU and anti-H3P revealed an increase in the number of EdU+/H3P+ double-positive cells in *hippo (RNAi)* animals versus controls (Fig 2B and S3B Fig). This suggests that in *hippo (RNAi)* animals, cells either transition faster from S to M phase and/or fail to complete M phase. The higher number of EdU-/H3P+ cells in *hippo (RNAi)* animals versus controls (Fig 2C and S3B Fig) indicates that the former group harbors a greater number of cells that are in M phase but have not passed through S phase in the previous 16 h. Thus, *hippo* RNAi results in the trapping of cells in M phase but does not affect the number entering the cell cycle (Fig 2D). This could explain why the increase in the number of H3P+ cells does not translate to an increase in cell number. However, we cannot rule out the possibility that *hippo* inhibition results in faster transit of cells from S to M phase.

To further understand the role of *hippo* in the cell cycle, we examined the organization of the mitotic spindle and chromosomes in dissociated cells from *hippo (RNAi)* animals by double immunostaining with anti-α-tubulin and anti-H3P antibodies. The results revealed abnormal microtubule organization of prophase/prometaphase spindles and spindle poles in *hippo (RNAi)* cells (Fig 2E). Similarly, metaphase cells showed disorganized and less dense arrays of spindle microtubules, as well as chromosome alignment defects (Fig 2E). Furthermore, in *hippo (RNAi)* cells, more than a half of all anaphases appeared in an early phase, while in controls, all were classified as late anaphases. Half of the early anaphases in *hippo (RNAi)* cells were characterized by a disorganized and less dense microtubule array (Fig 2E). This result supports an essential role of *hippo* in the normal cell cycle progression and is consistent with the increase in the number of cells in M phase, but not S phase, observed after *hippo* RNAi.

In agreement with the role of Hippo in cell cycle progression and specifically in the mitotic phase, we found that several genes related to cytokinesis and mitotic spindle organization were differentially expressed in the *hippo (RNAi)* RNA sequencing analysis (RNAseq) (S1 Table). Importantly, these included genes already known to be regulated by Mst1/2 or Lats1/2 and mainly involved in spindle orientation (*afadin, Drosophila discs large, polo-like kinase 1*) [38–40] (S1 Table).

### Hippo is required for maintenance of the differentiated cell population

**Fig 3:**
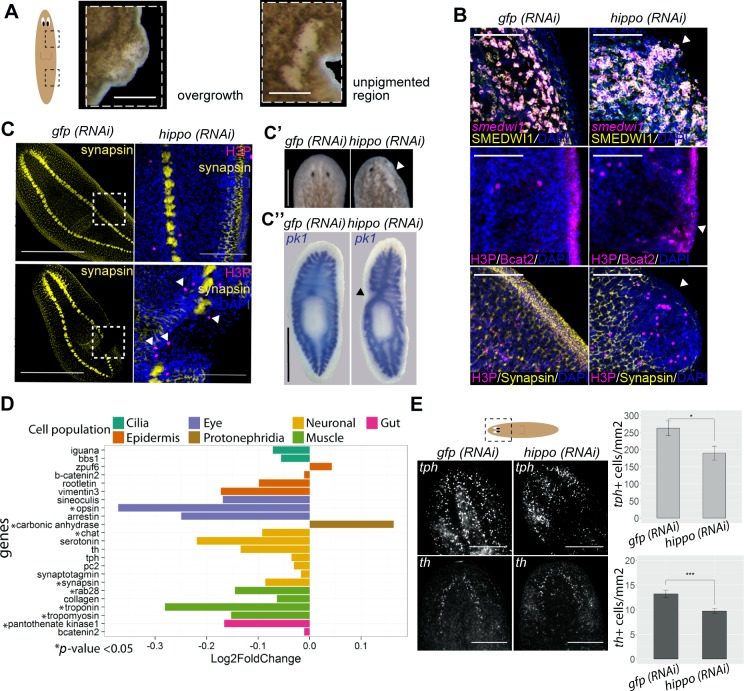
Hippo inhibition gives rise to overgrowths and extensive areas of undifferentiated cells. (A) Stereomicroscopic view of an overgrowth and an unpigmented region formed after *hippo* inhibition. (B) Analysis of overgrowths. From top to bottom: FISH combined with immunostaining to visualize *smedwi-1* mRNA and SMEDWI-1+ protein localization (arrow indicates the accumulation of undifferentiated cells in overgrowths); double immunostaining with anti-H3P and anti-β-catenin-2 antibodies to visualize mitosis and epidermal cells (arrow indicates the loss of β-catenin-2 staining in epidermal cells of the overgrowths); double immunostaining with anti-H3P and anti-synapsin antibodies (arrow indicates the loss of synapsin and the increase in mitotic cells in the overgrowths). Nuclei are labeled with DAPI. Images correspond to confocal Z-projections. (C) Analysis of the unpigmented regions. Anti-synapsin immunostaining of an unpigmented region in *hippo (RNAi)* animals. White box represents the magnified region. A detail of an unpigmented region showing double immunostaining with anti-synapsin and anti-H3P antibodies is shown. Nuclei are labeled with DAPI. Images correspond to confocal Z-projections. White arrows indicate H3P+ cells surrounding the undifferentiated region. (C′) Stereomicroscopic view of the anterior region showing the disappearance of anterior structures in *hippo (RNAi)* animals. White arrow indicates the region from which anterior structures disappear. (C″) In situ hybridization for *pk1* showing disappearance of the gut in an unpigmented region after *hippo* inhibition. Black arrow indicates region lacking a digestive system. (D) Graph showing the relative expression levels of cell markers for differentiated cells in the RNA sequence of *hippo (RNAi)* animals. (E) FISH staining of *tph* and *th* and the corresponding quantification. The total number of tph+ and th+ cells in the head region was normalized with respect to the total area of each animal. Images correspond to confocal Z-projections; *n* ≥ 5. All experiments were performed in planarians subjected to 3 weeks of *hippo* RNAi. Error bars represent standard deviation. Data were analyzed by Student *t* test. *p < 0.05; ***p < 0.001. Data used in the generation of this figure can be found in S1 Data. Scale bars: 150 μm (A, B, and C′); 1 mm (C and C″); and 200 μm (E). Bcat2, β-catenin-2; FISH, fluorescent in situ hybridization; *gfp*, *green fluorescent protein*; H3P, phospho-histone-H3-Ser10; *pk1*, *pantothenate kinase 1*; RNAi, RNA interference; *th*, *tyrosine hydroxylase* (dopaminergic neuron); *tph*, *tryptophan hydroxylase* (serotonergic neuron).

Detailed analysis of the overgrowths caused by *hippo* inhibition (Fig 3A) revealed that they are caused by the accumulation of cells in the subepidermal region or the mesenchyme (Fig 3B and S4A Fig) and in some cases arise from the submuscular plexus region (S1–S3 Movies). In line with the unpigmented appearance (Fig 3A), which indicates that epidermal cells cannot produce pigment and thus are not terminally differentiated, we observed abnormal distribution of β-catenin-2, a component of adherens junctions, in the epidermal cells of the overgrowths [41] (Fig 3B, S1–S3 Movies). The defects in cell differentiation also affected the neural plexus, as evidenced by the absence of anti-synapsin staining in the overgrown areas (Fig 3B). The accumulation of *smedwi-1+*/ SMEDWI-1+ cells in subepidermal overgrowths and among some cells of the corresponding epidermis (Fig 3B and S4A Fig) indicates that the overgrowths are primarily composed of undifferentiated cells. The accumulation of mitotic cells in or around the overgrowths (Fig 3B and S1–S3 Movies) confirms loss of the differentiated state.

In addition to the specific overgrown regions, broader unpigmented areas were also observed in *hippo (RNAi)* animals (Fig 3A). Analysis with specific markers revealed that differentiated structures such as the nervous system, the eyes, and the digestive system were not properly renewed or maintained in those unpigmented areas (Fig 3C–3C” and S4B Fig). Furthermore, *hippo (RNAi)* animals had a poorly developed digestive system, smaller pharynx and eyes (which, in some cases, were almost absent), and smaller brains (S4B Fig), consistent with general defects in the maintenance of differentiated structures. Notably, the smaller brains appeared to be surrounded by ectopic mitotic cells (S4B Fig). Analysis of the *hippo (RNAi)* RNAseq further indicated that most of the markers associated with terminal differentiation of multiple cell types (*opsin*, *pantothenate kinase 1*, *synapsin*, *tropomyosin*) were down-regulated (Fig 3D), consistent with a general defect in the process of cell differentiation or maintenance of the differentiated state. Quantification of the number of dopaminergic and serotonergic neurons by ISH with the corresponding markers (*tyrosine hydroxylase* and *tryptophan hydroxylase 1*) further confirmed the general decrease in the number of differentiated neurons in *hippo (RNAi)* planarian heads (Fig 3E).

In several animals, the disappearance of differentiated structures coincided with the presence of cell-depleted regions (Fig 3B and 3C). Therefore, using in vivo propidium iodide (PI) incorporation analysis, we investigated whether a general increase in cell death occurred in *hippo (RNAi)* animals. The results revealed an increase in PI+ cells in *hippo (RNAi)* animals (S5 Fig). Because we previously showed that caspase-dependent cell death is decreased in *hippo (RNAi)* animals, we can conclude that necrotic cell death is increased. This increase in necrosis could compensate for the decrease in apoptosis and contribute to the maintenance of cell number in *hippo (RNAi)* animals.

Taken together, these data indicate that Hippo plays an important role in the maintenance or renewal of differentiated tissues in planarians and that its inhibition leads to the appearance of extensive regions of undifferentiated cells that accumulate in overgrowths.

### *Hippo-Yorkie* signaling controls cell differentiation during planarian regeneration

**Fig 4:**
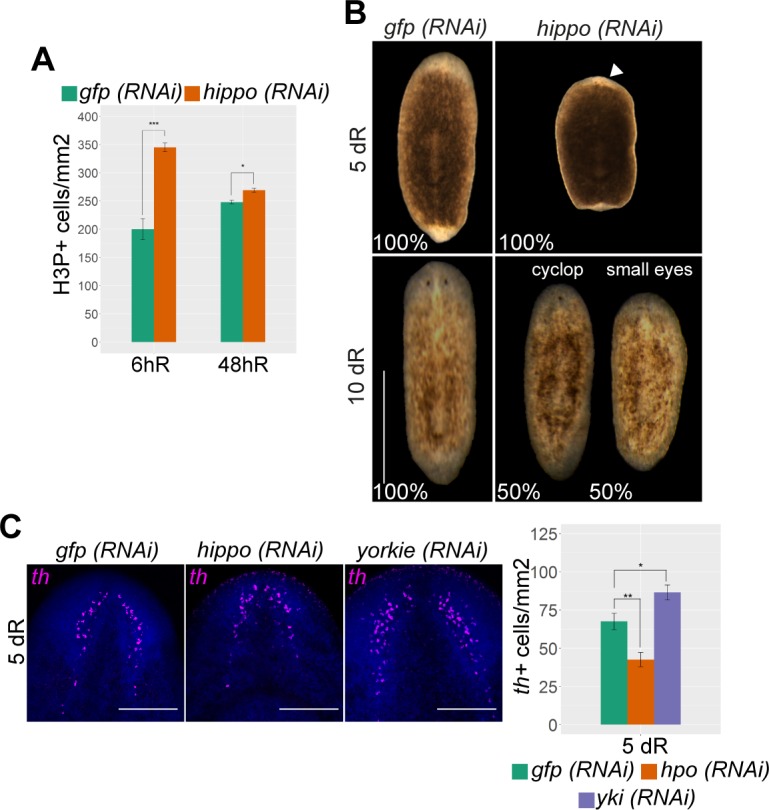
A *hippo-yki* signal is essential for proper planarian regeneration. (A) Quantification of H3P+ cells after 6 and 48 hRs in *hippo (RNAi)* animals and corresponding controls; *n* ≥ 8. (B) Stereomicroscopic view of live control and *hippo (RNAi)* animals after 5–10 dRs; *n* = 10. White arrow indicates the smaller blastema. (C) FISH for *th*+ in control, *hpo (RNAi)*, and *yki (RNAi)* animals after 5 dRs. Nuclei are stained with DAPI. The corresponding quantification is shown (*n* ≥ 5). The total number of *th*+ cells was normalized with respect to the head area (from the anterior tip to the pharynx). Error bars represent standard deviation. Data were analyzed by Student *t* test. *p < 0.05; **p < 0.01. Data used in the generation of this figure can be found in S1 Data. Scale bars: 500 μm (B); 150 μm (C). dR, days of regeneration; FISH, fluorescent in situ hybridization; *gfp*, *green fluorescent protein*; *hpo*, *hippo*; hR, hours of regeneration; H3P, phospho-histone-H3-Ser10; RNAi, RNA interference; *th*+, *tyrosine hydroxylase* (dopaminergic neuron); *yki*, *yorkie*.

In regenerative contexts, Yki/YAP nuclearization induced after Hippo inhibition or direct Yki/YAP overactivation is associated with increased proliferation and improved regenerative capacity [17], [18]. Analysis of the effect of *hippo* inhibition following head and tail amputation (S6A Fig) revealed an increase in mitotic rate but the production of smaller blastemas (Fig 4A and 4B), in which new tissues such as the brain and digestive system failed to properly differentiate (S6B and S6C Fig). Accordingly, numbers of differentiated neural cell types, such as dopaminergic neurons (*th+)* and eye cells (*ovo+* cells in the eye) [42] were reduced after *hippo* inhibition (Fig 4C and S6D Fig). Interestingly, despite the increase in mitotic rates and the defects in differentiation, overgrowths were never observed in regenerating *hippo (RNAi)* animals.

Although Yki/YAP is a highly evolutionarily conserved downstream element of the Hippo pathway [5], it has been proposed that Yki does not act downstream of Hippo in planarians [34]. To investigate the possible conservation of Hippo-Yki signaling in the control of planarian cell differentiation, we generated a specific anti-Yorkie antibody to determine the levels and pattern of Yki expression. Both western blot and immunohistochemical analysis revealed decreases in Yki levels in *yki (RNAi)* animals, as expected (S7 Fig). By contrast, in *hippo (RNAi)* planarians, Yki protein levels were specifically up-regulated in the nucleus (S7 Fig), suggesting conservation of a Hippo-Yki signal in planarians. We next investigated whether *yki* plays a role in cell differentiation in planarians. Inhibition of *yki* in regenerating animals led to an increase in the numbers of differentiated neurons and photoreceptors (Fig 4C and S6D Fig), in direct opposition to the phenotype observed following *hippo* inhibition. In agreement with our results, a recent study [43] reported a general increase in the numbers of several types of differentiated cells in *yki (RNAi)* planarians. Taken together, these results indicate that a conserved Hippo-Yki signal regulates cell differentiation during planarian regeneration. In contrast to the effect observed in vertebrate systems [17,18], in planarians, *hippo* inhibition, and hence Yki nuclearization, blocks differentiation and decreases the regenerative response.

### *hippo* defines the expression boundaries of epidermal markers

**Fig 5:**
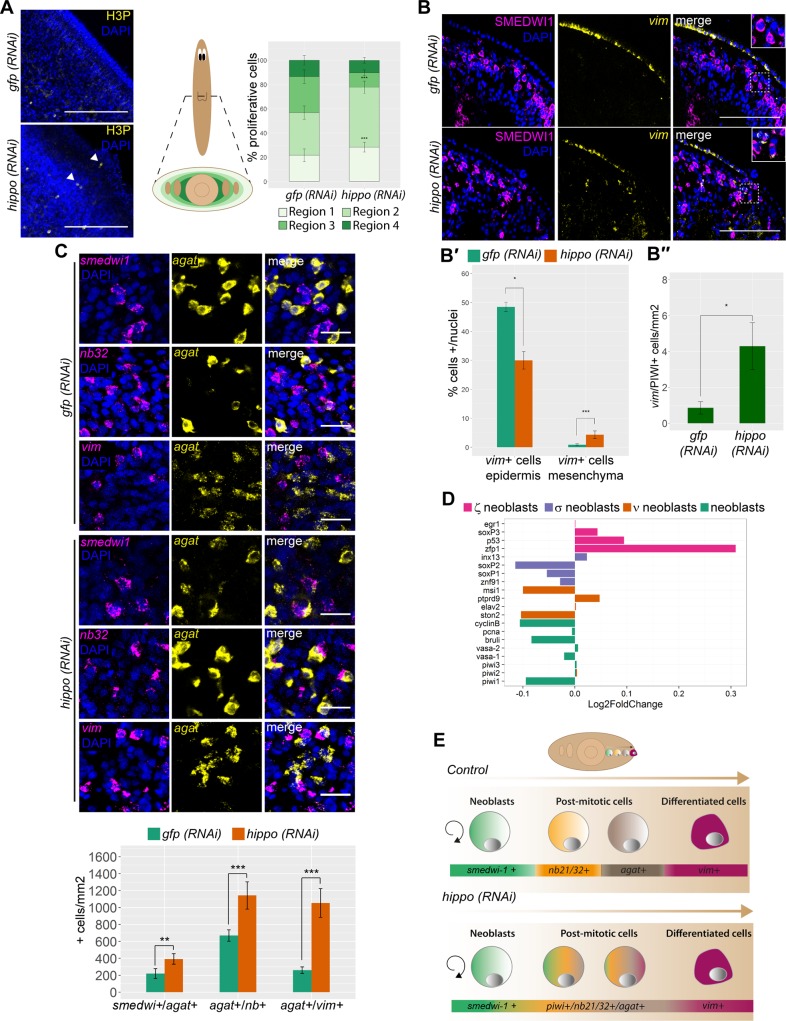
*hippo* defines the expression boundaries of epidermal markers. (A) Immunostaining with anti-H3P in the subepidermal region of hippo *(RNAi)* animals and controls. Nuclei are labeled in blue. A schematic indicating the different regions quantified, from outermost (light green) to innermost (dark green), is shown, as well as corresponding quantification of the number of H3P+ cells in the different regions along the mediolateral axis (*n* ≥ 6). The total number of H3P+ cells found in each region was normalized to the selected area, as indicated in the schematic. White arrows indicate H3P+ cells. (B) FISH for *vim* combined with anti-SMEDWI-1 immunostaining in transverse sections from *hippo (RNAi)* animals and controls. (B′) Corresponding quantification of *vim*+ cells in the epidermis and mesenchyme; *n* ≥ 6. (B″) Corresponding quantification of *vim*+/SMEDWI-1+ cells (*n* ≥ 6). (C) Double FISH with markers of different epidermal cell progenitors: *smedwi-1/agat*, *agat/nb32*, and *agat/vim* and corresponding quantification (*n* ≥ 6). (D) Graph showing the relative expression levels of different neoblast markers in the RNAseq of *hippo (RNAi)* animals [78, 79]. Error bars represent standard deviation. Data were analyzed by Student *t* test. *p < 0.05; **p < 0.01; ***p < 0.001. (E) Schematic showing the consequences of *hippo* inhibition in the epidermal lineage. In wild-type animals, the onset of stem cell differentiation results in the loss of *smedwi-1* expression and the acquisition of postmitotic genes in a sequential manner (*nb32 → agat → vim*) to reach the fully differentiated state in the epidermis (*vim*+ cells). In *hippo (RNAi)* animals, the spatiotemporal transition of postmitotic epidermal cells is lost: *smedwi-1*, *nb32*, *agat*, and *vim* are co-expressed in the same cells and the number of fully differentiated cells is decreased. All experiments were performed in planarians subjected to *hippo RNAi* for 3 weeks. Quantifications in (B) and (C) correspond to the posterior half of the body of each animal. Data used in the generation of this figure can be found in S1 Data. Scale bars: 150 μm (A); 50 μm (B); 25 μm (C). FISH, fluorescent in situ hybridization; *gfp*, *green fluorescent protein*; H3P, phospho-histone-H3-Ser10; RNAi, RNA interference; *vim*, *vimentin*.

The epidermal lineage is the most abundant in planarians and its progression and determination is well understood [44]. Epidermal maturation requires temporally correlated transition states in planarians, in which stem cells (*smedwi-1+*) become postmitotic and start to sequentially express *nb21/32*, *agat*, and *vimentin* (*vim*) [44,45]. In parallel, epidermal precursors migrate from the inner parenchyma towards the epidermis [44]. Thus, proliferating cells are mainly found in the inner part of the animal and postmitotic epidermal cells are found at the periphery. Interestingly, in *hippo (RNAi)* planarians we detected a large number of mitotic cells in the periphery, where overgrowths and unpigmented regions were mainly found (Fig 5A). Because this region should mainly contain postmitotic epidermal precursors [44], we reasoned that the process of differentiation and/or cell fate maintenance of the epidermal lineage may be impaired in *hippo (RNAi)* animals. In agreement with this hypothesis, we found that *hippo* was expressed in all epidermal lineage cells (S1A Fig and S8A Fig).

To gain further insight into cell fate progression in *hippo (RNAi)* animals, we analyzed the number and distribution of mitotic cells (H3P+), stem cells (*smedwi-1+*), and postmitotic epidermal cells (*nb21/32+*, *agat+*, or *vim+*). First, we quantified H3P+ cells in 4 different regions (inner to outermost) in transverse sections (Fig 5A). We observed a significant increase in the number of mitotic cells (H3P+) in the 2 outermost regions of *hippo (RNAi)* animals as compared with controls (S5A and S8B Figs). *vim*+ cells were located mainly in the epidermis (fully differentiated cells) of control animals but were predominantly detected in the mesenchyme of *hippo (RNAi)* animals (Fig 5B and 5B′). This indicates that *hippo* RNAi impairs the acquisition or maintenance of epithelial fate or the migration of epidermal cells. We detected higher numbers of *vim+*/ SMEDWI-1+ cells in the mesenchyme of *hippo (RNAi)* animals versus control animals, in which these cells were virtually absent from the mesenchyme (Fig 5B–5B″). This finding, together with the presence of ectopic mitotic cells, suggests impairment of the differentiation or fate maintenance, but not the migration, of epidermal cells following *hippo* inhibition.

Quantification of cells that were double positive for different epidermal markers revealed an increase in *smedwi-1*+/*agat*+, *nb*+/*agat*+, and *agat*+/*vim*+ cells in *hippo (RNAi)* animals versus controls (Fig 5C). This increase in bivalent cells in *hippo (RNAi)* animals is consistent with failure of the epidermal cells to progress appropriately through the hierarchical transitions that occur during epidermal lineage specification and suggests that they are unable to maintain a defined fate. Interestingly, double labeling of H3P+/*agat*+, H3P+/*nb32*+, or H3P+/*smedwi-1*+ revealed that mitotic cells were always *smedwi-1*+ but never *agat*+ nor *nb32*+ in either *hippo (RNAi)* animals or controls (S8C Fig). Although we cannot exclude the possibility that ectopic mitotic cells could arise from a different lineage or from aberrant migration of stem cells, our findings suggest that mitotic cells never express postmitotic markers.

Supporting the view that *hippo* inhibition interferes with specification of the epidermal lineage, analysis of the *hippo (RNAi)* RNA sequence revealed an increase in the expression of markers of ζ-neoblasts (*zfp-1* and *p53*), which are precursors of the epidermal lineage [31] (Fig 5D). This increase could be caused by an increase in the number of ζ-neoblasts. However, we found that while expression levels of *smedwi-1*, a general marker of stem cells, decreased in the transcriptome (Fig 5D), the number of cells expressing this marker was unchanged in *hippo (RNAi)* animals (S8D Fig). As such, the increased expression of ζ-neoblast markers could also be due to increased expression of those markers in ζ-neoblasts or to incorrect acquisition of their expression by other cell types.

Taken together, our analysis of the epidermal lineage reveals an increase in the population of cells co-expressing cell markers that define different epidermal cell lineages in *hippo (RNAi)* animals, as well as mislocalization of these markers (Fig 5E). Thus, precursors and differentiated epidermal cells are both improperly defined and mislocalized. These results indicate that *hippo* is essential for the acquisition and/or maintenance of cell fate in the epidermal lineage.

### The Hippo pathway maintains the differentiated state in planarians

**Fig 6:**
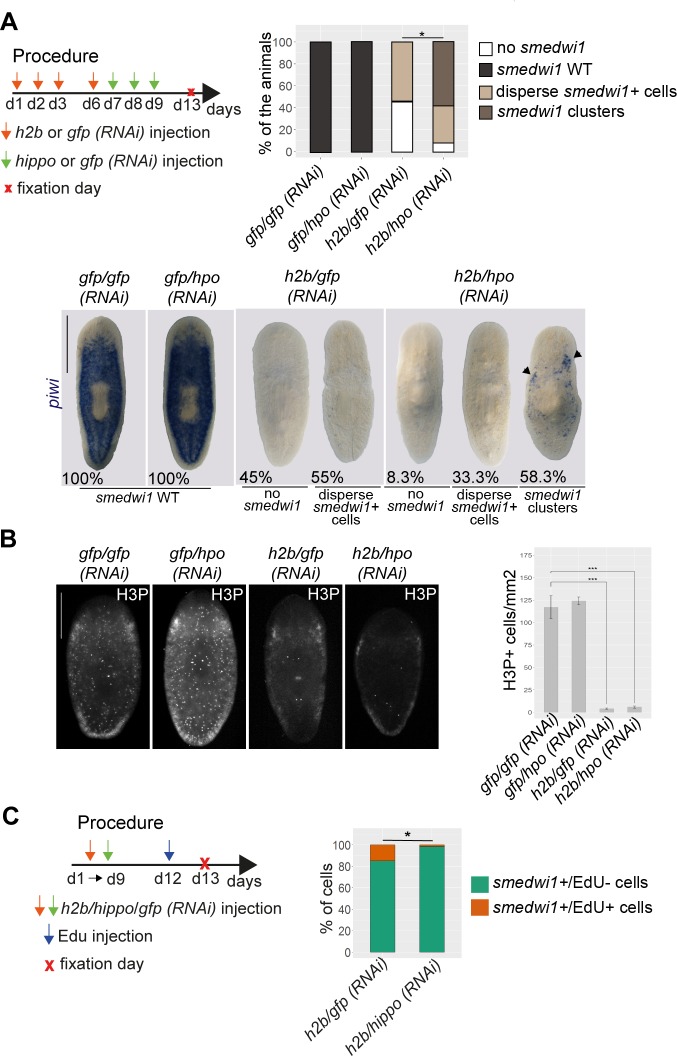
*hippo* maintains the differentiated state in planarians. (A) Cartoon illustrating the experimental design of the *hippo* RNAi experiment in neoblast-depleted animals. ISH for *smedwi-1* in the 4 RNAi conditions and corresponding quantification of *smedwi-1* expression distribution and classification in 4 categories (*n* ≥ 10). Black arrows indicate *smedwi-1+* clusters. Data were analyzed by chi-squared test, applying a Bonferroni correction (*p < 0.01). (B) Immunostaining of *h2b/hippo (RNAi)* animals and corresponding controls with anti-H3P antibody, and corresponding quantification. The total number of H3P+ cells in the whole animal was quantified and normalized with respect to total body area. Error bars represent standard deviation (*n* ≥ 14). Data were analyzed using a Student *t* test (***p < 0.001). Scale bars: 1 mm (A). (C) EdU incorporation followed by FISH of *smedwi-1* in *h2b (RNAi)* and *h2b/hippo (RNAi)* animals. The procedure used and the corresponding quantification of *smedwi-1+/*EdU- and *smedwi-1+/*Edu+ cells are shown. In total, 15 sections corresponding to *n* ≥ 8 animals were analyzed. Data were analyzed by chi-squared test, applying a Bonferroni correction (*p < 0.01). Data used in the generation of this figure can be found in S1 Data. EdU, 5-ethynyl-2′-deoxyuridine; FISH, fluorescent in situ hybridization; *gfp*, green fluorescent protein; *hpo*, *hippo*; H3P, phospho-histone-H3-Ser10; ISH, in situ hybridization; RNAi, RNA interference.

The presence of large numbers of undifferentiated cells and overgrowths following *hippo* RNAi could be explained either by defective differentiation of stem cells towards a specific fate or by a dedifferentiation process in which postmitotic cells are unable to maintain the committed state and begin to express stem-cell markers. To investigate the possibility that postmitotic cells undergo dedifferentiation, we depleted the stem-cell population in planarians through X-ray irradiation followed by the inhibition of *hippo* over 4 consecutive days (S9A Fig). ISH for *smedwi-1* revealed that after lethal irradiation (60 Gy) no *smedwi-1+* cells were detectable in either controls or *hippo (RNAi)* animals (S9B Fig). However, in planarians that received irradiation at sublethal doses (17.5 Gy), *hippo* inhibition increased the number of *smedwi-1+* cells (S9C Fig). Importantly, anti-H3P immunostaining revealed no difference in the number of mitotic cells between sublethally irradiated/*hippo (RNAi)* planarians and their corresponding controls, indicating that the increase in *smedwi-1+* cells cannot be attributed to increased proliferation (S9D Fig). Because DNA damage induced by high doses of irradiation could mask the possible effect of *hippo* inhibition on cell dedifferentiation, we next investigated the effects of *histone 2b* (*h2b*) inhibition as an alternative means of depleting the neoblast population [46], followed by *hippo RNAi* (Fig 6A). qPCR analysis using specific primers for *h2b* and *hippo* confirmed inhibition of both genes (S10A Fig). ISH for *smedwi-1* revealed no differences between *hippo (RNAi)* animals and controls (the “*smedwi-1* WT” phenotype in Fig 6A), whereas in *h2b (RNAi)* animals, *smedwi-1* expression was completely absent (“no *smedwi-1*” phenotype in Fig 6A) (45%), or reduced to a few scattered cells (“disperse *smedwi-1*+ cells” phenotype in Fig 6A) (55%), which corresponded to stem cells that escaped the effects of *h2b* inhibition (Fig 6A). Strikingly, several animals in the *h2b/hippo (RNAi)* group had clusters of *smedwi-1*+ cells in the marginal region of the body (“*smedwi-1* clusters” phenotype in Fig 6A) (58.3%) (Fig 6A). Interestingly, this corresponded to the region in which ectopic mitosis and overgrowths were observed. Moreover, despite the presence of *smedwi-1* clusters, the number of H3P+ cells in *h2b/hippo (RNAi)* animals was comparable to that of *h2b (RNAi)* animals and was much lower than that of controls (Fig 6B). In addition, EdU incorporation analysis revealed that the percentage of *smedwi-1+* cells that entered the cell cycle in *h2b/hippo (RNAi)* animals was lower than that of *h2b (RNAi)* animals (Fig 6C and S10B Fig). This result supports the view that *smedwi-1+* cells originate from distinct populations in *h2b (RNAi)* and *h2b/hippo (RNAi)* animals. Taken together, our data suggest that the clusters of *smedwi-1+* cells found in *h2b/hippo (RNAi)* animals are not the result of the proliferation of remaining stem cells that escape *h2b* inhibition but are produced by dedifferentiation of postmitotic cells that regain the expression of stem-cell markers.

To better understand the nature of *smedwi-1+* cells in *h2b/hippo (RNAi)* animals, we investigated whether the expression of different neoblast markers was also increased. The results revealed that expression of the stem-cell marker *bruli* [47] was also increased, although not to the same degree as *smedwi-1* (S10C Fig). Because our previous results showed that *hippo* is required to properly maintain epidermal fate and that the expression of ζ-neoblast markers is increased after *hippo* RNAi, we analyzed the *smedwi-1+* clusters observed in *h2b/hippo (RNAi)* animals for expression of the ζ-neoblast marker *zfp1*. ISH showed that *h2b/hippo (RNAi)* animals were mostly depleted of *zfp1*, as were *h2b (RNAi)* animals (S10D Fig). This result does not allow us to determine whether *smedwi-1+* clusters arise from the dedifferentiation of epidermal cells. It is possible that *smedwi-1+* clusters arise from a different lineage or, alternatively, that lineage restriction is not maintained during dedifferentiation of epidermal cells. Nonetheless, our results suggest that Hippo is essential to maintain the postmitotic state in planarians and that its absence may induce the dedifferentiation of postmitotic cells towards a stem-cell identity.

## Discussion

In the present study, we show that *hippo* is required to regulate the apoptotic and mitotic response in starved degrowing planarians. *hippo RNAi* resulted in an increase in the number of mitotic cells and a decrease in the number of apoptotic cells. Importantly, and in contrast to reports in other organisms [2,48,49], the imbalance between apoptosis and mitosis observed following *hippo* inhibition did not lead to changes in planarian body size or cell number. This observation may be explained by the increase in the number of necrotic cells after *hippo* inhibition. Furthermore, the fact that the increase in the number of cells in M phase in *hippo (RNAi)* animals was not accompanied by an increase in the number of cells in S phase may also contribute to the maintenance of the cell number in *hippo (RNAi)* planarians. Our findings demonstrate that in planarians, Hippo is essential for proper cell cycle progression and, specifically, for successful completion of mitosis. The role of core Hippo signaling elements in critical mitotic events, namely in centrosomal duplication, chromosomal alignment, spindle formation, and completion of cytokinesis, has also been reported in *Drosophila* and vertebrate species [36,40,50–53]. Planarian *hippo (RNAi)* cells showed improper assembly of the mitotic spindle. This effect, together with the number of genes directly involved in mitosis that are differentially regulated in the *hippo (RNAi)* transcriptome, suggests a direct role of Hippo in regulating their expression. For instance, centriolin, which is required to complete cytokinesis, was down-regulated in the *hippo (RNAi)* transcriptome. Corroborating the important role of Hippo in the cell cycle in planarians, in the transcriptome we detected several genes previously reported to mediate the requirement of Hippo for proper cell cycle progression (S1 Table). Despite defective mitotic exit, *hippo (RNAi)* animals showed no tissue regression, the phenotype normally associated with loss of the cycling cell population in planarians [29]. Indeed, the total population of stem cells or neoblasts (*smedwi-1+* cells) was maintained in *hippo (RNAi)* animals. This suggests that only a fraction of the cells that enter the cell cycle become arrested in M phase after *hippo* inhibition. Supporting this view, around 30% of mitotic figures in *hippo (RNAi)* cells were defective.

There is a second possible explanation for the observation that the imbalance between apoptosis and mitosis in *hippo (RNAi)* animals does not lead to changes in total cell number: a fraction of the cells found in M phase could arise from the dedifferentiation of postmitotic cells. Supporting this hypothesis, in *hippo (RNAi)* planarians we detected ectopic mitotic cells in the periphery, a region in which postmitotic cells are normally located. These mitotic cells also exhibited aberrant co-expression of committed epidermal cell markers and therefore are unlikely to be the result of incorrect migration of stem cells, which are normally located in the innermost part of the organism. Moreover, the boundaries of expression of the epidermal markers corresponding to the entire lineage were not conserved, indicating that *hippo* inhibition impairs the maintenance of cell identity. The poorly committed cells found in *hippo (RNAi)* animals could arise during the process of differentiation or could be the product of induced cell dedifferentiation. Importantly, we found that *hippo* inhibition in planarians depleted of cycling cells (or neoblasts) may promote dedifferentiation of postmitotic cells. It should be stressed that although a process of cell dedifferentiation that allows re-entry in the cell cycle is common in regenerative models, it has never been described in animals with the level of plasticity found in planarians, which fulfill their regenerative and renewal requirements by activating proliferation of their abundant population of totipotent stem cells [33, 54]. Thus, our data strongly indicate that *hippo* plays a crucial role in the maintenance of the differentiated state and that its inhibition promotes dedifferentiation.

Hippo inhibition in planarians promotes the formation of overgrowths, as described in all models in which it has been studied to date [1,48,55]. Although early studies of the Hippo pathway in model organisms attributed the appearance of tumors to an imbalance between cell death and proliferation [1,55], our results indicate that the inability of cells to maintain the differentiated state following *hippo* inhibition may underlie the appearance of undifferentiated regions and the formation of overgrowths, which also consisted of undifferentiated cells. In agreement with this hypothesis, the formation of overgrowths was preceded by the appearance of large areas in which different tissues, including those pertaining to the neural, digestive, and visual systems, showed a loss of differentiated cell types. Those results are in good agreement with recently published findings supporting an essential role of *hippo* in restricting cell plasticity in the liver and intestine [9,23], an effect that has been linked to its role in the recruitment of chromatin-remodeling complexes [14,56]. Thus, nuclear Yki/YAP, rather than pluripotency, could exert a critical role in conferring plasticity, which is a crucial property of tumor cells [57].

**Fig 7:**
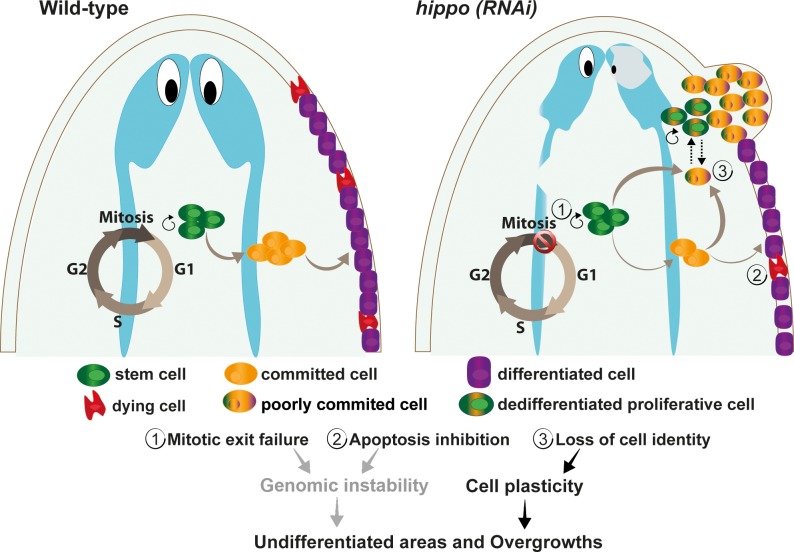
Model of the *hippo* function in planarians. In wild-type animals, stem cells or neoblasts can proliferate or can become committed and differentiate into specific cell types. Committed cells exit the cell cycle to proceed to the differentiation process. The health and number of postmitotic cells is also controlled by programmed cell death. In *hippo (RNAi)* planarians, proliferating stem cells or neoblasts become trapped in M phase (1), and fewer of these cells undergo apoptosis (2). Both alterations may lead to genomic instability, because the failure to exit M phase is linked to DNA damage, and inhibition of the apoptotic response hampers the elimination of damaged and old cells. *hippo* inhibition also promotes cell plasticity and facilitates cell dedifferentiation (3). Both genomic instability and cell plasticity could promote the appearance of undifferentiated areas and overgrowths. RNAi, RNA interference.

The formation of overgrowths could be due to the inhibition of apoptosis induced following *hippo* RNAi, in addition to the increase in cell plasticity (Fig 7). In wild-type animals, senescent cells and cells with DNA disarrangements are eliminated by apoptosis. However, these cells could not be eliminated in *hippo (RNAi)* animals and may contribute to the acquisition of additional genetic or epigenetic changes (Fig 7). Furthermore, the number of cells with DNA rearrangements and aneuploidies is probably increased in *hippo (RNAi)* planarians, because, according to our results, those animals display dysregulation of critical mitotic regulators and fail to complete cytokinesis, as previously demonstrated in LATS2 knockout mouse embryos [36]. Importantly, the control of apoptosis is intrinsically linked to genomic instability and malignant transformation [58]. It must be noted that the cell death that is inhibited after *hippo* inhibition in our experimental conditions is the one induced by starvation. However, it is likely that the damage-induced apoptosis also depends on *hippo*, because both starvation- and damage-induced apoptosis depend on such common mechanisms as JNK activation [59].

Taking into account the location of the overgrowths in the subepidermal region, which is abundant in epidermal precursors and is also the location of ectopic mitotic cells, it is tempting to speculate that the epidermal lineage is the origin of the cell accumulations in *hippo (RNAi)* animals. Epidermal cell plasticity promoted by *hippo* inhibition could account for the appearance of bivalent cells that are susceptible to tumoral transformation. Furthermore, although the total number of stem cells was not altered after *hippo* inhibition, we observed a marked increase in the expression of markers of ζ-neoblasts, the precursors of epidermal cells, underscoring the important role of the pathway in restricting the fate of this specific lineage. However, the fact that *hippo* inhibition in planarians depleted of cycling cells (or neoblasts) resulted in the formation of *smedwi-1+* cell clusters that did not express the ζ-neoblast marker *zfp1* does not support an epidermal origin. A deeper analysis of the molecular features of these *smedwi-1+* cells will be required to determine whether they arise from a nonepidermal cell type or, alternatively, whether dedifferentiation keeps the cells in a more pluripotent state. Nonetheless, the fact that almost all markers of differentiation were down-regulated in the *hippo (RNAi)* transcriptome and the appearance of undifferentiated areas affecting neuronal, epithelial, and digestive cells in *hippo (RNAi)* animals indicate that *hippo* controls the fate of several cell types and that different cell lineages could contribute to the overgrowths observed. To understand whether *hippo* exerts a predominant role in specific tissues, it will be necessary to determine whether precursor cell markers are also aberrantly co-expressed following *hippo* inhibition in other cell types.

In addition to its role in maintaining cell fate, *hippo* may also be required for proper cell differentiation, as reported in other organisms [10,13,14]. In our study, the role of *hippo* in cell differentiation was more evident in the context of regeneration, because the decrease in the number of differentiated cells observed in newly regenerating blastemas, despite the increase in mitotic cells, could not directly result from dedifferentiation. Moreover, the observed defects in differentiation may be caused by the dedifferentiation of postmitotic cells in pre-existing tissue after *hippo* RNAi, as previously described in intact animals, leading to misexpression by differentiated muscle cells of signaling molecules required for proper differentiation [60]. This possibility is consistent with the phenotype generated after *yki* inhibition in planarians, in which an increase in signaling molecules and differentiated cells is observed [43] (Fig 4C and S6 Fig).

The results presented here support an essential role for *hippo* in the acquisition and maintenance of differentiated cell fates. Thus, as demonstrated in other models, Hippo favors stemness in planarians. Importantly, although planarians possess an abundant population of totipotent stem cells, *hippo* inhibition promotes stemness not by increasing stem cell renewal and expansion of the stem cell compartment but rather by promoting cell plasticity and thus dedifferentiation of postmitotic cells. This finding has profound implications in the fields of regenerative medicine and cancer therapy, because the acquisition of plasticity by lineage-committed cells favors wound healing but also promotes tumorigenesis [28,61]. Accordingly, the cell plasticity induced by *hippo* inhibition is associated with beneficial effects in the context of heart and liver regeneration in vertebrates [17,18]. However, *hippo* inhibition in planarians, which have a much greater regenerative ability than vertebrates, impairs rather than promotes regeneration. One explanation for these contrasting effects is that in vertebrate regenerative systems, a process of cell dedifferentiation that allows re-entry in the cell cycle fuels the regenerative response [62,63], whereas in planarians, expansion of the stem cell population is the only source of new cells [33]. In homeostatic conditions, when basal cell renewal is required, *hippo* inhibition is also deleterious in planarians, because tumoral transformation is facilitated by the sustained increase in cell plasticity and the probable chromosomal instability induced by the cell cycle defects and the inhibition of apoptosis. The fact that we observed no overgrowths in regenerating regions further underscores the importance of the sustained effect of *hippo* inhibition in specific cell types to promote transformation.

## Conclusion

The present findings demonstrate that in a stem cell–based system such as the planarian, the main role of the Hippo pathway is not to control the balance between proliferating and dying cells nor to regulate body size or the stem-cell population; in planarians, *hippo* is required to successfully complete the cell cycle and to promote apoptosis. Furthermore, *hippo* is necessary to acquire and maintain cell fate, thus restricting cell plasticity. Consequently, long-term *hippo* inhibition prevents tissue renewal and leads to the formation of overgrowths.

Although further evidence is required, our results suggest that in planarians, as described in other organisms, *hippo* may be involved in maintenance of the chromatin state and the genome stability of stem cells and postmitotic descendants. This function would explain the systematic alteration of *hippo* signaling elements in many types of cancer, as well as the proregenerative effect of the induction of Yki nuclearization. However, it remains unclear why *hippo* elements are mutated in most human tumors but never at their onset, whereas in model organisms, the appearance of overgrowths is a consistent feature of *hippo* inhibition. Finally, our results highlight the potentially hazardous effects of manipulating the *hippo* pathway for medical purposes in regenerative medicine if the stem cells and progenitors induced after *hippo* inhibition are in fact poorly committed cells.

## Materials and methods

### Planarian culture

Asexual planarians from a clonal strain of *S*. *mediterranea* BCN-10 were maintained at 20°C in planarian artificial medium (PAM) water, as previously described [64]. Animals were fed with veal liver and starved for at least 1 week before beginning experiments.

### Cloning of *hippo* and *yki*

To amplify the *hippo* fragment, we used the follow primers: 5′-CGAGCACTGTTTATGATTCCTTC-3′ and 5′-CTCGGCTTGCAAGTCTGAGTC-3′. To amplify the *yorkie* fragment, the following primers were used: 5′-GTTTGGATGAATTATTCGAAGTGG-3′ and 5′-CACAATACAAAAGAAACCACATGG-3′. *hippo* and *yorkie* PCR fragments were cloned into pCRII (Life Technologies) and pGEM-T Easy (Promega) vectors to synthesize dsRNA or ssRNA, as required.

### RNA interference analysis

dsRNA was synthetized by in vitro transcription (Roche) and microinjection performed, as previously described [65], following the standard protocol of 3 × 32 nl injection of dsRNA for 3 consecutive days. To achieve stronger *hippo* inhibition, we performed the same protocol over 3 consecutive weeks in starved planarians. *yorkie (RNAi)* animals were injected for only 1 week with injection of *gfp* during the 2 previous weeks. Regenerating animals were injected with *hippo* or *yorkie* dsRNA for 2 weeks prior to amputation of the head and tail. Animals were fixed at different times postamputation, depending on the experiment. Control animals were injected with dsRNA for green fluorescent protein (GFP), a gene not present in planarians.

In the *h2b/hippo* experiment, animals were injected with *gfp* or *h2b* on 3 consecutive days and, after 2 rest days, received another injection of *gfp* or *h2b*. Beginning the following day, animals were injected with *gfp* or *hippo* for 3 consecutive days. Animals were fixed 4 days after the last injection.

### Quantitative real-time PCR

Total RNA was extracted from a pool of 5 planarians each for *hippo* RNAi and *gfp* RNAi. Quantitative real-time PCR was performed as previously described [46], and data were normalized based on the expression of EF2 or URA4 as an internal control. All experiments were performed using 3 biological replicates. The following sets of specific primers were used: hippo mRNA, 5′-TTTGGTCTTTGGGAATCAC-3′ and 5′-TGGAGGAGGTTGAGAAGG-3′; h2b mRNA, 5′-GAGAAAGTTGAACGGCCC-3′ and 5′-AAGATAATACGTACTTCAACGACG-3′.

### Whole-mount ISH and immunohistochemistry

RNA probes were synthesized in vitro using Sp6 or T7 polymerase (Roche) and DIG-, FITC-, or DNP-modified (Perkin Elmer) nucleotides. RNA probes were purified and precipitated with ethanol and 7.5 M ammonium acetate. For ISH and fluorescent in situ hybridization (FISH), animals were fixed and processed as previously described [66,67]. After probe development, neoblasts were visualized with the rabbit anti-SMEDWI-1 antibody (1:1,000; kindly provided by Kerstin Bartscherer, Max Plank Institute for Molecular Biomedicine, Münster, Germany) [47]. Nuclei were stained with DAPI (1:5,000) and mounted with 70% glycerol in PBS.

### Whole-mount immunostaining

Immunostaining was performed as previously described [68]. The following antibodies were used: mouse anti-synapsin (anti-SYNORF-1, 1:50; Hybridoma Bank); rabbit anti-phospho-histone-H3-Ser10 (anti-H3P) (1:500; Cell Signaling Technology); rabbit anti-SMEDWI-1 antibody (1:1,000); mouse anti-arrestin (anti-VC1) (1:15,000; kindly provided by Professor K. Watanabe); rabbit anti-β-catenin-2 (anti-Bcat2) (1:2,000) [41]. Nuclei were stained with DAPI (1:5,000) and mounted with 70% glycerol in PBS. To avoid technical variance and a reliable quantification of H3P+cells, at least 2 independent experiments were performed.

### ISH and immunohistochemistry in paraffin sections

For ISH and immunohistochemistry, animals were killed and processed as previously described [69]. The antibodies used were rabbit anti-SMEDWI-1 (1:1,000), rabbit anti-phospho-histone-H3-Ser10 (anti-H3P) (1:500; Cell Signaling Technology), rat anti-phospho-histone-H3-Ser10 (anti- H3P) (1:1,000; Millipore), and rabbit anti-YORKIE (1:200).

### Immunohistochemistry in dissociated cells

Whole animals were macerated in a solution containing methanol: glacial acetic acid: glycerol: distilled water (3:1:2:14) for 16 h at 4°C. Cells were transferred onto a slide and immunostained with mouse anti-α-Tubulin (1:500, Sigma) and rabbit anti-phospho-histone-H3-Ser10 (anti- H3P) (1:500; Cell Signaling Technology).

### Whole-mount TUNEL and caspase-3 activity assay

For TUNEL analysis, animals were fixed and treated as previously described [70], using the ApopTag Red In Situ Apoptosis Detection Kit (Merck-Millipore Ref.S7165). To avoid technical variance, at least 2 independent TUNEL experiments were performed. For the caspase-3 assay, total protein was extracted from a pool of 5 planarians and processed as previously described [71]. Enzyme activity was measured in a luminescence spectrophotometer (Perkin-Elmer LS-50) (1 excitation, 380 nm). A unit of caspase-3 activity was defined as the amount of active enzyme necessary to produce an increase of 1 arbitrary luminescence unit after a 2-h incubation. The results are presented as units of caspase-3 activity per μg of protein. All experiments were performed using 5 biological and 3 technical replicates for each condition. To avoid technical variance, at least 2 independent experiments were performed.

### Flow cytometry

Dissociation of planarians, cell labeling, and isolation of cells by FACS were performed as described previously [72]. Absolute cell counts were performed by adding beads of a known concentration to the sample. Beads and cells were detected simultaneously, and absolute counts of the cells were calculated from bead numbers (absolute counts by indirect method). Flow-Check Fluorospheres Polystyrene Fluorescent Microspheres (Beckman Coulter Inc, Indianapolis, IN) were adjusted at 1 × 10^6^ fluorospheres/mL. A 1:100 dilution of the bead solution was applied to each sample to obtain a final concentration of 1 × 10^4^ beads/mL. Beads and cells were identified according to their distinct patterns of scatter and fluorescence.

### Edu staining of paraffin sections

F-ara Edu (32 nl; Sigma) was injected into *gfp* and *hippo (RNAi)* animals at a concentration of 60 μg/mL (diluted in 10% DMSO/PAM water). After 16 h, animals were processed and stained with the EdU Click-555 kit (Baseclick Gmbh; BCK-Edu555), in accordance with the manufacturer’s recommendations, following pretreatment with proteinase K (20 μg/mL) for 10 min at RT. Samples were immunostained with anti-H3P, stained with DAPI, and mounted with 70% glycerol in PBS. In the *h2b/hippo (RNAi)* experiment, animals previously injected with gfp/h2b and/or gfp/hippo were injected with EdU and after 16 h were processed for FISH using the *smedwi-1* probe, followed by EdU staining.

### In vivo PI incorporation analysis

Animals were injected with *gfp* or *hippo* over 3 consecutive weeks and then injected with 3 × 32 nl of a mixture of PI (1.66 × 10^−3^ μg/μL; Sigma) and Hoechst 33342 (3.3 × 10^−6^ μg/μL; Sigma) diluted in PAM water. Immediately, planarians were soaked in the same solution for 4 h. Single planarians were placed on a microscopy slide and enveloped in a drop of 3% low melting agarose. Confocal imaging was performed within minutes of preparation of the samples.

### Generation of the anti-YORKIE polyclonal antibody

The complete coding sequence of *yorkie* cDNA was cloned into a p-GEMT Easy vector (Promega). Subcloning, protein expression, and antibody production were performed using ProteoGenix (ProteoGenix, France). Briefly, 200 μg of recombinant protein was used as an immunogen to produce polyclonal IgGs in 2 rabbits. The postimmunization serum was purified using protein A affinity purification, precipitated in sodium azide buffer, and stored at 4°C.

### Western blot assay

Protein extracts were obtained as previously described [73]. After incubation with rabbit anti-YORKIE (1:2,000) and mouse anti-α-TUBULIN (1:10,000; Sigma) antibodies, the signal was developed using Clarity Western ECL Substrate (Bio-Rad) and chemiluminescence was detected using a C-DiGit Chemiluminescent Western Blot Scanner (LI-COR). Quantifications were performed with Image Studio Lite and normalized to the anti-α-Tubulin signal.

### Transcriptomic analysis

For transcriptomic analysis, total RNA was extracted from *gfp (RNAi)* and *hippo (RNAi)* animals after 1, 3, or 4 weeks of inhibition. Three replicates were generated per condition from a pool of 5 organisms each. RNA was extracted with Trizol (Invitrogen), following the manufacturer's instructions. RNA was quantified with a Nanodrop ND-1000 spectrophotometer (Thermo Scientific) and quality assessment performed by capillary electrophoresis in an Agilent 2100 Bioanalyzer (Agilent Technologies) prior to preparation of the library. cDNA libraries were prepared using the Illumina TruSeq Stranded mRNA Library Prep Kit and sequenced as paired-end reads in an Illlumina HiSeq 2000 sequencer. Quality assessment of the reads was performed using the FastQC suite. Transcript abundances were calculated with kallisto v0.43.0 [74] on the *S*. *mediterranea* dd_Smed_v6 transcriptome assembly [75]. Differential expression analysis was carried out using the sleuth [76] and DESeq2 [77] statistical packages. Raw sequencing data in FASTQ format as well as the transcript abundances have been deposited in the NCBI Gene Expression Omnibus (GEO) [78] and are accessible using GEO Series accession number GSE95130.

### Imaging

FISH and immunostaining samples were imaged using a MZ16F stereomicroscope (Leica) equipped with a ProgRes C3 camera (Jenoptik) or an SP2 confocal laser-scanning microscope (Leica). Images were processed using Fiji and Photoshop CS5 (Adobe) software. Brightness/contrast and color balance adjustments were always applied to the entire image. Quantifications were performed by hand using the “multi-point selection” tool of Fiji. Colocalization quantification was performed using the equivalent areas using the “ROI-manager” tool in Fiji. Nuclear area in Edu and IP experiments was measured using the “threshold” tool with the “moments” mask for all samples. Signal quantification of Yorkie antibody immunostaining was processed using Fiji software. Two planes were used to build the Z-projection. Nuclear-stained (DAPI) images were transformed into a mask using the “threshold” tool with the “moments” mask. The mask was used to obtain the nuclear signal with the Image calculator process. The nuclear signal obtained from the resulting image was measured to obtain the raw integrated density (RID). The nuclear area was obtained from the mask. Next, the RID was normalized to the respective nuclear area. Results were averaged per group and significant differences determined by 2-tailed Student *t* test.

## Supporting information

S1 Fig *Hippo* is ubiquitously expressed in planarians and *hippo* RNAi produces overgrowths. (A) *hippo* expression levels in different cell types according to a single-cell RNAseq analysis (74). (B) In situ hybridization for *hippo* reveals a ubiquitous expression pattern. (C) Cartoon illustrating the experimental design used for *hippo* RNAi during planarian homeostasis. Animals were starved for 1 week before the experiment and were then injected on 3 consecutive days each week for 3 weeks. Starvation was maintained throughout. (D) Relative expression levels of *hippo* after *hippo* RNAi, as measured by qRT-PCR. Values represent the means of 3 biological replicates. Error bars represent standard deviation. Data were analyzed by Student *t* test. *p < 0.05; **p < 0.01. (E) Graph showing the evolution of *hippo (RNAi)* phenotypes after 16, 17, and 18 days of *hippo* inhibition. Data used in the generation of this figure can be found in S1 Data. RNAi, RNA interference; RNAseq, RNA sequencing analysis.
(TIF)

S2 Fig *Hippo* controls both cell death and mitotic levels in planarians. (A) Whole-mount TUNEL showing apoptotic cell death in planarians subjected to *hippo* RNAi for 3 weeks (*n* ≥ 5). Images correspond to confocal Z-projections. (B) Quantification of caspase-3 activity after 1, 2, and 3 weeks of *hippo* inhibition. Results are presented as units of caspase-3 activity per μg of protein. Bars correspond to the mean of 3 biological replicates. Error bars represent standard deviation. (C) Immunostaining with anti-H3P antibody in planarians subjected to *hippo* RNAi for 3 weeks (*n* ≥ 10). (D) Graph showing the total cell number in planarians subjected to *hippo* RNAi for 3 weeks, as determined using a Neubauer chamber. Bars correspond to the mean of 3 biological replicates. Error bars represent standard deviation. Data were analyzed by Student *t* test. **p < 0.01; ***p < 0.001. Data used in the generation of this figure can be found in S1 Data. Scale bars: 250 μm (A); 1 mm (B). n.s., not significant; RNAi, RNA interference.
(TIF)

S3 Fig *Hippo* is essential for G2/M transition and M exit in planarians. (A) Cartoon illustrating the EdU pulse procedure. Animals were starved for 1 week, injected with *hippo* dsRNA for 3 weeks, and then injected with EdU and fixed 16 h later. (B) EdU labeling in transverse sections combined with immunostaining with anti-H3P antibody in the pharynx region in controls and in planarians subjected to *hippo* RNAi for 3 weeks. Scale bars: 50 μm. dsRNA, double-stranded RNA; EdU, 5-ethynyl-2′-deoxyuridine; H3P, phospho-histone-H3-Ser10; RNAi, RNA interference.
(TIF)

S4 Fig Cellular and molecular analysis of overgrowths and unpigmented regions in *hippo (RNAi)* animals. (A) Analysis of overgrowths. FISH combined with immunostaining showing the localization of *smedwi-1* mRNA and SMEDWI-1 protein. Colocalization of both signals appears to be concentrated in the overgrowths, indicating that they consist of undifferentiated cells. Arrowhead indicates an epidermal cell of an overgrowth stained with SMEDWI-1. (B) Analysis of unpigmented regions. Immunostaining using different markers. From left to right: staining of the epithelia with anti-anti-Bcat2 antibody; digestive system labeled with anti-Bcat2 antibody (white arrows indicate gut branches); pharynx labeled with anti-Bcat2 antibody; head region stained with anti-synapsin, anti-H3P, and anti-Bcat2 antibodies (arrowheads indicate mitotic cells); sagittal section showing a head region stained with anti-H3P (arrowheads indicate mitotic cells; discontinuous line delimits the brain); visual system stained with anti-arrestin (VC-1). Blue corresponds to nuclei stained with DAPI. All experiments were performed in planarians subjected to *hippo* RNAi for 3 weeks. All images correspond to confocal Z-projections. Scale bars: 100 μm; 200 μm (A); 100 μm; 250 μm; 150 μm; 250 μm; 150 μm; 100 μm (B). Bcat2, β-catenin-2; Br, brain; FISH, fluorescent in situ hybridization; H3P, phospho-histone-H3-Ser10; RNAi, RNA interference.
(TIF)

S5 Fig Inhibition of *hippo* increases in vivo PI incorporation. Staining of dead cells using PI in live control and *hippo (RNAi)* animals. Nuclei are stained with Hoechst. Magnifications of the indicated area are shown below. Arrowhead indicates some cells positive for PI. A stereomicroscopic view of live control and *hippo (RNAi)* animals used in the experiment is shown. Quantification of the PI+ cells per nuclei area in the head region is shown. Data were analyzed by Student *t* test (*n* = 4). ***p < 0.001. Data used in the generation of this figure can be found in S1 Data. Scale bars: 100 μm (top images); 25 μm (bottom images). PI, propidium iodide; RNAi, RNA interference.
(TIF)

S6 Fig A *hippo*-*yki* signal regulates cell differentiation during planarian regeneration. (A) Cartoon illustrating the RNAi procedure in regenerating conditions. Animals were starved for 1 week before the experiment and then injected on 3 consecutive days. The following week, animals were injected again on 3 consecutive days, cut the next day, and fixed at different time points. (B) Anti-synapsin immunostaining of the brain region of control and *hippo (RNAi)* animals. Nuclei are stained with DAPI. Images correspond to planarians after 12 dR. (C) In situ hybridization with *pk1* (digestive system) in *hippo (RNAi)* and control animals. Images correspond to planarians after 10 dR. (D) Fluorescent in situ hybridization for *ovo* to label eyes in *hippo (RNAi)*, *yorkie (RNAi)*, and control animals. Images correspond to planarians after 5 dR. The corresponding quantification is shown. Data were analyzed by Student *t* test. *p < 0.05. Data used in the generation of this figure can be found in S1 Data. Scale bars: 200 μm (B); 0.5 mm (C); 50 μm (D). dR, days of regeneration; *pk1*, *pantothenate kinase 1*; RNAi, RNA interference.
(TIF)

S7 Fig Yorkie may be one of the Hippo pathway effectors in planarians. (A) Western blot of protein extracts from *hippo (RNAi)*, *yorkie (RNAi)*, and control planarians immunoblotted with anti-Yorkie and anti-α-Tubulin antibodies. Extracts correspond to intact animals after 3 weeks (*hippo (RNAi)*) or 1 week (*yorkie (RNAi)*) of RNAi. The corresponding quantification of the Yorkie versus Tubulin signal is shown. Bars represent the mean of 3 biological replicates. (B) Immunostaining with anti-Yorkie in longitudinal sections of *hippo (RNAi)*, *yorkie (RNAi)*, and control planarians. *hippo* was inhibited for 3 consecutive weeks and *yorkie* for 1 week. Nuclei were stained with DAPI. Magnifications of the indicated region are included. The corresponding quantification of the nuclear signal in the post-pharyngeal region is shown. Yorkie nuclear signal intensity was measured as RID and then normalized to the corresponding nuclear area (see Materials and methods). Error bars represent standard deviation (*n* ≥ 3). Data were analyzed by Student *t* test. *p < 0.05; **p < 0.01. Data used in the generation of this figure can be found in S1 Data. Scale bars: 50 μm (B). RID, raw integrated density; RNAi, RNA interference.
(TIF)

S8 Fig Analysis of mitotic cells and epidermal lineage markers. (A) FISH showing colocalization of *hippo* with *smedwi-1*, *nb32*, and *agat*. (B) Immunostaining using anti-H3P antibody in transverse sections. Nuclei are stained with DAPI. White arrows indicate H3P+ cells. (C) FISH with *smedwi-1*, *nb32*, and *agat* combined with immunostaining with anti-H3P. Nuclei are stained with DAPI. (D) Graph showing the quantification of *smedwi-1*+ cells in control and *hippo (RNAi)* animals. *smedwi-1*+ cells were quantified in the posterior half and normalized with respect to the area quantified. Error bars represent the standard deviation (*n* ≥ 7). Data were analyzed by Student *t* test. Data used in the generation of this figure can be found in S1 Data. Scale bars: 15 μm (A); 100 μm (B); 25 μm (C). FISH, fluorescent in situ hybridization; H3P, phospho-histone-H3-Ser10; n.s., not significant; RNAi, RNA interference.
(TIF)

S9 Fig Analysis of X-ray-irradiated/*hippo(RNAi)* animals. (A) Cartoon illustrating the irradiation and dsRNA injection procedures. Animals were starved for 1 week, exposed to different doses of radiation (60 Gy and 17.5 Gy), and injected with *hippo* or *gfp* dsRNA for 4 consecutive days. Animals were fixed 7 days after the beginning of the experiment. (B) In situ hybridization for *smedwi-1* probe in animals irradiated with 60 Gy. No signal is detected (*n* ≥ 18). (C) In situ hybridization for *smedwi-1* in *gfp* and *hippo (RNAi)* animals irradiated with 17.5 Gy. Black arrows indicate *smedwi-1+* cells/clusters. The corresponding quantification of *smedwi-1* expression, distribution, and classification into 4 categories is shown (*n* ≥ 18). Data were analyzed by chi-squared test, applying a Bonferroni correction (*p < 0.05). (D) Anti-H3P immunostaining in *gfp* and *hippo (RNAi)* animals irradiated with 17.5 Gy. Corresponding quantification is shown. H3P+ cells in the whole animal were quantified and normalized to the total area of each animal. Error bars represent standard deviation (*n* ≥ 13). Data were analyzed by Student *t* test. Data used in the generation of this figure can be found in S1 Data. Scale bars: 1 mm. dsRNA, double-stranded RNA; *gfp*, *green fluorescent protein*; H3P, phospho-histone-H3-Ser10; n.s., not significant; RNAi, RNA interference.
(TIF)

S10 Fig Molecular analysis of *h2b/hippo (RNAi)* animals. (A) Relative expression of *h2b* and *hippo*, as determined by qRT-PCR, in planarians 4 days postinjection. Values represent the means of 3 biological replicates. Error bars represent standard deviation. Data were analyzed by Student *t* test. *p < 0.05; ***p < 0.001. (B) EdU labeling in transverse paraffin sections combined with immunostaining with anti-H3P antibody in *h2b/gfp (RNAi)* and *h2b/hippo (RNAi)* animals. Arrowhead indicates *smedwi-1+* cells, some of which are EdU+. Fifteen sections corresponding to *n* ≥ 8 animals were analyzed. (C) In situ hybridization for *bruli* in *h2b/hippo (RNAi)* animals and corresponding controls. Black arrows indicate *bruli+* cells. Quantification of *bruli* expression and classification into 2 categories (*n* ≥ 6). Data were analyzed by chi-squared test, applying a Bonferroni correction; *p < 0.05. (D) In situ hybridization for *zfp*1 in the 4 RNAi conditions, corresponding quantification of *zfp1* expression, and classification into 4 categories (*n* ≥ 10). Data were analyzed by chi-squared test, applying a Bonferroni correction. Data used in the generation of this figure can be found in S1 Data. Scale bars: 15 μm (B); 1 mm (C and D). EdU, 5-ethynyl-2′-deoxyuridine; *h2b*, *histone 2b*; H3P, phospho-histone-H3-Ser10; RNAi, RNA interference.
(TIF)

S1 Movie Bcat2 staining of a planarian following *hippo* RNAi. Movie showing anti-Bcat2 immunostaining of a region corresponding to an overgrowth formed following *hippo* inhibition. Nuclei are labeled with DAPI. Anti-Bcat2 antibody nonspecifically labels adherens junctions in epithelial cells and muscle fibers. Accumulation of nuclei can be observed from the innermost section, corresponding to the area above the muscle plexus, to the outer sections, corresponding to the outermost part of the animal (i.e., the epidermis). The cell–cell junctions of epidermal cells appear disrupted in *(RNAi)* animals (yellow arrow). In the inner sections, accumulation of cells can be observed among the muscle tissue (white arrow). Bcat2, β-catenin-2; RNAi, RNA interference.
(AVI)

S2 Movie Bcat2 and H3P staining of a *hippo (RNAi)* planarian. Movie showing anti-Bcat2 and anti-H3P immunostaining of a region corresponding to an overgrowth in a *hippo (RNAi)* animal. Both antibodies are shown in red. The H3P signal but not the Bcat2 signal colocalizes with nuclear DAPI staining. Anti-Bcat2 nonspecifically labels adherens junctions in epithelial cells and muscle fibers. Accumulation of H3P+ nuclei is observed in the marginal portion of the planarian. In the overgrown area, epidermal integrity is maintained despite the loss of Bcat2 signal (which labels adherens junctions) (yellow arrow). Note the appearance of accumulated cells in the muscle layer (white arrow). Bcat2, β-catenin-2; RNAi, RNA interference.
(AVI)

S3 Movie Bcat2 and H3P staining in a control planarian. Movie showing anti-Bcat2 and H3P immunostaining in a control animal. Both antibodies are shown in red. The H3P signal but not the Bcat2 signal colocalizes with nuclear DAPI staining. Anti-Bcat2 nonspecifically labels adherens junctions in epithelial cells and muscle fibers. H3P+ nuclei are observed in the innermost sections. Staining of adherens junctions with Bcat2 antibody is evident in the epidermis. Bcat2, β-catenin-2.
(AVI)

S1 Table Up- and down-regulation in the *hippo (RNAi)* RNAseq of genes involved in the cell cycle. Selected up- or down-regulated genes in the RNAseq of *hippo (RNAi)* animals involved in cell cycle. Each column contains the following information: PlanMine code (74), gene name, gene symbol, E-val (indicating homology with the predicted gene), fold change, adjusted *p*-value, gene function, and bibliography. Studies demonstrating the role of each gene in the cell cycle or its relation with Hippo pathway are cited. The *p*-value is adjusted to an FDR of 0.05. FDR, false discovery rate; RNAi, RNA interference; RNAseq, RNA sequencing analysis.
(XLSX)

S1 Data All individual numerical values that underlie the summary data shown in the following figures: Fig 1B, 1C, 1D and 1E; Fig 2A, 2B and 2C; Fig 3E; Fig 4A and 4C; Fig 5A, 5B′, 5B″ and 5C; Fig 6A, 6B and 6C; S1D and S1E Fig; S2B and S2D Fig; S5 Fig; S6D Fig; S7A and S7B Fig; S8D Fig; S9C and S9D Fig; and S10A, S10C and S10D Fig. (XLSX)

## Abbreviations

dR: days of regeneration
dsRNA: double-stranded RNA
EdU: 5-ethynyl-2′-deoxyuridine
ES: embryonic stem
FACS: fluorescence-activated cell sorting
FISH: fluorescent in situ hybridization
GEO: Gene Expression Omnibus
*gfp*: *green fluorescent protein*
*hpo*: *hippo*
hR: hours of regeneration
*h2b*: *histone 2b*
H3P: phospho-histone-H3-Ser10
iPS: induced pluripotent stem
ISH: in situ hybridization
n.s.: not significant
PAM: planarian artificial medium
PI: propidium iodide
*pk1*: pantothenate kinase 1
RID: raw integrated density
RNAi: RNA interference
RNAseq: RNA sequencing analysis
*th*: *tyrosine hydroxylase*
*tph*: *tryptophan hydroxylase*
*vim*: *vimentin*
Yki: Yorkie

## References

[1] Udan R. S., Kango-Singh M., Nolo R., Tao C., and Halder G., “Hippo promotes proliferation arrest and apoptosis in the Salvador/Warts pathway.,” Nat. Cell Biol., vol. 5, no. 10, pp. 914–920, 2003 doi: 10.1038/ncb1050 14502294 10.1038/ncb1050

[2] Camargo F. D., Gokhale S., Johnnidis J. B., Fu D., Bell G. W., Jaenisch R., and Brummelkamp T. R., “YAP1 Increases Organ Size and Expands Undifferentiated Progenitor Cells,” Curr. Biol., vol. 17, no. 23, pp. 2054–2060, 2007 doi: 10.1016/j.cub.2007.10.039 17980593 10.1016/j.cub.2007.10.039

[3] Wu S., Huang J., Dong J., and Pan D., “hippo encodes a Ste-20 family protein kinase that restricts cell proliferation and promotes apoptosis in conjunction with salvador and warts,” Cell, vol. 114, no. 4, pp. 445–456, 2003 12941273 10.1016/s0092-8674(03)00549-x

[4] Harvey K. F., Pfleger C. M., and Hariharan I. K., “The Drosophila Mst ortholog, hippo, restricts growth and cell proliferation and promotes apoptosis,” Cell, vol. 114, no. 4, pp. 457–467, 2003 12941274 10.1016/s0092-8674(03)00557-9

[5] Huang J., Wu S., Barrera J., Matthews K., and Pan D., “The Hippo signaling pathway coordinately regulates cell proliferation and apoptosis by inactivating Yorkie, the Drosophila homolog of YAP,” Cell, vol. 122, no. 3, pp. 421–434, 2005 doi: 10.1016/j.cell.2005.06.007 16096061 10.1016/j.cell.2005.06.007

[6] Cao X., Cao X., Pfaff S. L., Pfaff S. L., Gage F. H., and Gage F. H., “YAP regulates neural progenitor cell number via the TEA domain transcription factor,” Genes \& Dev., vol. 22, no. 23, p. 3320, 2008. 19015275 10.1101/gad.1726608 PMC2600760

[7] Karpowicz P., Perez J., and Perrimon N., “The Hippo tumor suppressor pathway regulates intestinal stem cell regeneration.,” Development, vol. 137, no. 24, pp. 4135–4145, 2010 doi: 10.1242/dev.060483 21098564 10.1242/dev.060483 PMC2990205

[8] Ramos A. and Camargo F. D., “The Hippo signaling pathway and stem cell biology,” Trends Cell Biol., vol. 22, no. 7, pp. 339–346, 2012 doi: 10.1016/j.tcb.2012.04.006 22658639 10.1016/j.tcb.2012.04.006 PMC3383919

[9] Yimlamai D., Christodoulou C., Galli G. G., Yanger K., Pepe-Mooney B., Gurung B., Shrestha K., Cahan P., Stanger B. Z., and Camargo F. D., “Hippo pathway activity influences liver cell fate,” Cell, vol. 157, no. 6, pp. 1324–1338, 2014 doi: 10.1016/j.cell.2014.03.060 24906150 10.1016/j.cell.2014.03.060 PMC4136468

[10] Mahoney J. E., Mori M., Szymaniak A. D., Varelas X., and Cardoso W. V., “The Hippo Pathway Effector Yap Controls Patterning and Differentiation of Airway Epithelial Progenitors,” Dev. Cell, vol. 30, no. 2, pp. 137–150, 2014 doi: 10.1016/j.devcel.2014.06.003 25043473 10.1016/j.devcel.2014.06.003 PMC6331061

[11] Harvey K. and Tapon N., “The Salvador-Warts-Hippo pathway an emerging tumour-suppressor network,” Nat Rev Cancer, vol. 7, no. 3, pp. 182–191, 2007 doi: 10.1038/nrc2070 17318211 10.1038/nrc2070

[12] Zhao B., Wei X., Li W., Udan R. S., Yang Q., Kim J., Xie J., Ikenoue T., Yu J., Li L., Zheng P., Ye K., Chinnaiyan A., Halder G., Lai Z., and Guan K.-L., “Inactivation of YAP oncoprotein by the Hippo pathway is involved in cell contact inhibition and tissue growth control,” Genes Dev., vol. 21, no. 21, pp. 2747–2761, 2007 doi: 10.1101/gad.1602907 17974916 10.1101/gad.1602907 PMC2045129

[13] J.-H. et al. Hong, “TAZ, a Transcriptional Modulator of Mesenchymal Stem Cell Differentiation,” Science *(80-**)*, vol. 309, no. 5737, pp. 1074–1078, 2005 doi: 10.1126/science.1110955 16099986 10.1126/science.1110955

[14] Aylon Y., Sarver A., Tovy A., Ainbinder E., and Oren M., “Lats2 is critical for the pluripotency and proper differentiation of stem cells,” Cell Death Differ., vol. 21, no. 10, pp. 624–633, 2014. 24413153 10.1038/cdd.2013.188 PMC3950325

[15] Hao Y., Chun A., Cheung K., Rashidi B., and Yang X., “Tumor suppressor LATS1 is a negative regulator of oncogene YAP,” J. Biol. Chem., vol. 283, no. 9, pp. 5496–5509, 2007 doi: 10.1074/jbc.M709037200 18158288 10.1074/jbc.M709037200

[16] Moroishi T., Hansen C. G., and Guan K.-L., “The emerging roles of YAP and TAZ in cancer.,” Nat. Rev. Cancer, vol. 15, no. 2, pp. 73–9, 2015 doi: 10.1038/nrc3876 25592648 10.1038/nrc3876 PMC4562315

[17] Zhou Q., Li L., Zhao B., and Guan K., “The Hippo pathway in heart development, regeneration, and diseases.,” Circ. Res., vol. 116, no. 8, pp. 1431–47, 2015 doi: 10.1161/CIRCRESAHA.116.303311 25858067 10.1161/CIRCRESAHA.116.303311 PMC4394208

[18] Loforese G., Malinka T., Keogh A., Baier F., Simillion C., Montani M., Halazonetis T. D., Candinas D., and Stroka D., “Impaired liver regeneration in aged mice can be rescued by silencing Hippo core kinases MST1 and MST2.,” EMBO Mol. Med., pp. 1–15, 2016 doi: 10.15252/emmm.201505943 27940445 10.15252/emmm.201506089 PMC5210079

[19] Ren F., Wang B., Yue T., Yun E.-Y., Ip Y. T., and Jiang J., “Hippo signaling regulates Drosophila intestine stem cell proliferation through multiple pathways.,” Pnas, vol. 107, no. 49, pp. 21064–21069, 2010 doi: 10.1073/pnas.1012759107 21078993 10.1073/pnas.1012759107 PMC3000252

[20] Zhang H., Pasolli H. A., and Fuchs E., “Yes-associated protein (YAP) transcriptional coactivator functions in balancing growth and differentiation in skin,” Proc Natl Acad Sci U S A, vol. 108, no. 6, pp. 2270–2275, 2011 doi: 10.1073/pnas.1019603108 21262812 10.1073/pnas.1019603108 PMC3038759

[21] Heallen T., Zhang M., Wang J., Bonilla-Claudio M., Klysik E., Johnson R. L., and Martin J. F., “Hippo Pathway Inhibits Wnt Signaling to Restrain Cardiomyocyte Proliferation and Heart Size,” Science *(80-**)*, vol. 332, no. 6028, pp. 458–461, 2011 doi: 10.1126/science.1199010 21512031 10.1126/science.1199010 PMC3133743

[22] Bai H., Zhang N., Xu Y., Chen Q., Khan M., Potter J. J., Nayar S. K., Cornish T., Alpini G., Bronk S., Pan D., and Anders R. A., “Yes-associated protein regulates the hepatic response after bile duct ligation,” Hepatology, vol. 56, no. 3, pp. 1097–1107, 2012 doi: 10.1002/hep.25769 22886419 10.1002/hep.25769 PMC3431197

[23] Gregorieff A., Liu Y., Inanlou M. R., Khomchuk Y., and Wrana J. L., “Yap-dependent reprogramming of Lgr5+ stem cells drives intestinal regeneration and cancer,” Nature, vol. 526, no. 7575, pp. 715–718, 2015 doi: 10.1038/nature15382 26503053 10.1038/nature15382

[24] Qin H., Blaschke K., Wei G., Ohi Y., Blouin L., Qi Z., Yu J., Yeh R. F., Hebrok M., and Ramalho-santos M., “Transcriptional analysis of pluripotency reveals the hippo pathway as a barrier to reprogramming,” Hum. Mol. Genet., vol. 21, no. 9, pp. 2054–2067, 2012 doi: 10.1093/hmg/dds023 22286172 10.1093/hmg/dds023 PMC3315209

[25] Panciera T., Azzolin L., Fujimura A., Di Biagio D., Frasson C., Bresolin S., Soligo S., Basso G., Bicciato S., Rosato A., Cordenonsi M., and Piccolo S., “Induction of Expandable Tissue-Specific Stem/Progenitor Cells through Transient Expression of YAP/TAZ,” Cell Stem Cell, vol. 19, no. 6, pp. 725–737, 2016 doi: 10.1016/j.stem.2016.08.009 27641305 10.1016/j.stem.2016.08.009 PMC5145813

[26] A. As. and Yamanaka S., “Rethinking differentiation: Stem cells, regeneration, and plasticity,” Cell, vol. 157, no. 1, pp. 110–119, 2014 doi: 10.1016/j.cell.2014.02.041 24679530 10.1016/j.cell.2014.02.041 PMC4074550

[27] Hogan B. L. M., Barkauskas C. E., Chapman H. A., Epstein J. A., Jain R., Hsia C. C. W., Niklason L., Calle E., Le A., Randell S. H., Rock J., Snitow M., Krummel M., Stripp B. R., Vu T., White E. S., Whitsett J. A., and Morrisey E. E., “Repair and regeneration of the respiratory system: Complexity, plasticity, and mechanisms of lung stem cell function,” Cell Stem Cell, vol. 15, no. 2, pp. 123–138, 2014 doi: 10.1016/j.stem.2014.07.012 25105578 10.1016/j.stem.2014.07.012 PMC4212493

[28] Vicente-Dueñas C., Gutiérrez de Diego J., Rodríguez F. D., Jiménez R., and Cobaleda C., “The role of cellular plasticity in cancer development.,” Curr. Med. Chem., vol. 16, no. 28, pp. 3676–85, 2009 19747147 10.2174/092986709789105019

[29] Reddien P. W., “SMEDWI-2 Is a PIWI-Like Protein That Regulates Planarian Stem Cells,” Science *(80-**)*, vol. 310, no. 5752, pp. 1327–1330, 2005 doi: 10.1126/science.1116110 16311336 10.1126/science.1116110

[30] Wagner D. E., Wang I. E., and Reddien P. W., “Clonogenic Neoblasts Are Pluripotent Adult Stem Cells That Underlie Planarian Regeneration,” Science *(80-**)*, vol. 811, no. 2011, pp. 1–48, 2011. 10.1126/science.1203983 PMC3338249 21566185

[31] Van Wolfswinkel J. C., Wagner D. E., and Reddien P. W., “Single-cell analysis reveals functionally distinct classes within the planarian stem cell compartment,” Cell Stem Cell, vol. 15, no. 3, pp. 326–339, 2014 doi: 10.1016/j.stem.2014.06.007 25017721 10.1016/j.stem.2014.06.007 PMC4171737

[32] Scimone M. L., Kravarik K. M., Lapan S. W., and Reddien P. W., “Neoblast specialization in regeneration of the planarian schmidtea mediterranea,” Stem Cell Reports, vol. 3, no. 2, pp. 339–352, 2014 doi: 10.1016/j.stemcr.2014.06.001 25254346 10.1016/j.stemcr.2014.06.001 PMC4176530

[33] Saló E. and Baguñà J., “Regeneration and pattern formation in planarians. II. Local Origin and Role of Cell Movements in Blastema Formation,” Development, vol. 107, pp. 69–76, 1989.

[34] Lin A. Y. T. and Pearson B. J., “Planarian yorkie/YAP functions to integrate adult stem cell proliferation, organ homeostasis and maintenance of axial patterning,” Development, vol. 141, no. 6, pp. 1197–1208, 2014 doi: 10.1242/dev.101915 24523458 10.1242/dev.101915

[35] Wurtzel O., Cote L. E., Poirier A., Satija R., Regev A., and Reddien P. W., “A Generic and Cell-Type-Specific Wound Response Precedes Regeneration in Planarians,” Dev. Cell, vol. 35, no. 5, pp. 632–645, 2015 doi: 10.1016/j.devcel.2015.11.004 26651295 10.1016/j.devcel.2015.11.004 PMC4817857

[36] Yabuta N., Okada N., Ito A., Hosomi T., Nishihara S., Sasayama Y., Fujimori A., Okuzaki D., Zhao H., Ikawa M., Okabe M., and Nojima H., “Lats2 is an essential mitotic regulator required for the coordination of cell division,” J. Biol. Chem., vol. 282, no. 26, pp. 19259–19271, 2007 doi: 10.1074/jbc.M608562200 17478426 10.1074/jbc.M608562200

[37] Hergovich A. and Hemmings B. A., “Hippo signalling in the G2/M cell cycle phase: Lessons learned from the yeast MEN and SIN pathways,” Semin. Cell Dev. Biol., vol. 23, no. 7, pp. 794–802, 2012 doi: 10.1016/j.semcdb.2012.04.001 22525225 10.1016/j.semcdb.2012.04.001 PMC3459816

[38] Rakotomamonjy J., Brunner M., Jüschke C., Zang K., Huang E. J., Reichardt L. F., and Chenn A., “Afadin controls cell polarization and mitotic spindle orientation in developing cortical radial glia,” Neural Dev., vol. 12, no. 1, p. 7, 2017 doi: 10.1186/s13064-017-0085-2 28482867 10.1186/s13064-017-0085-2 PMC5422985

[39] Keder A., Rives-Quinto N., Aerne B. L., Franco M., Tapon N., and Carmena A., “The Hippo pathway core cassette regulates asymmetric cell division,” Curr. Biol., vol. 25, no. 21, pp. 2739–2750, 2015 doi: 10.1016/j.cub.2015.08.064 26592338 10.1016/j.cub.2015.08.064

[40] Hergovich A., “Hippo Signaling in Mitosis: An Updated View in Light of the MEN Pathway,” Mitotic Exit Netw. Methods Protoc. Methods Mol. Biol., vol. 1505, pp. 265–277, 2017. 10.1007/978-1-4939-6502-1_19 27826870

[41] Chai G., Ma C., Bao K., Zheng L., Wang X., Sun Z., Salò E., Adell T., and Wu W., “Complete functional segregation of planarian β-catenin-1 and -2 in mediating Wnt signaling and cell adhesion,” J. Biol. Chem., vol. 285, no. 31, pp. 24120–24130, 2010 doi: 10.1074/jbc.M110.113662 20511647 10.1074/jbc.M110.113662 PMC2911336

[42] Lapan S. W. and Reddien P. W., “Transcriptome Analysis of the Planarian Eye Identifies ovo as a Specific Regulator of Eye Regeneration,” Cell Rep., vol. 2, no. 2, pp. 294–307, 2012 doi: 10.1016/j.celrep.2012.06.018 22884275 10.1016/j.celrep.2012.06.018 PMC3785364

[43] Lin A. Y. T. and Pearson B. J., “Yorkie is required to restrict the injury responses in planarians,” PLoS Biol., vol. 13, no. 7, pp. 1–30, 2017. 10.1371/journal.pgen.1006874 PMC5515462 28686611

[44] Eisenhoffer G. T., Kang H., and Alvarado A. S., “Molecular Analysis of Stem Cells and Their Descendants during Cell Turnover and Regeneration in the Planarian Schmidtea mediterranea,” Cell Stem Cell, vol. 3, no. 3, pp. 327–339, 2008 doi: 10.1016/j.stem.2008.07.002 18786419 10.1016/j.stem.2008.07.002 PMC2614339

[45] Tu K. C., Cheng L. C., Vu H. T. K., Lange J. J., McKinney S. A., Seidel C. W., and Sánchez Alvarado A., “Egr-5 is a post-mitotic regulator of planarian epidermal differentiation,” Elife, vol. 4, no. 12, pp. 1–27, 2015. 10.7554/eLife.10501 PMC4716842 26457503

[46] Solana J., Kao D., Mihaylova Y., Jaber-Hijazi F., Malla S., Wilson R., and Aboobaker A., “Defining the molecular profile of planarian pluripotent stem cells using a combinatorial RNAseq, RNA interference and irradiation approach.,” Genome Biol., vol. 13, no. 3, pp. 1–23, 2012. 10.1186/gb-2012-13-3-r19 PMC3439970 22439894

[47] Guo T., Peters A. H. F. M., and Newmark P. A., “A bruno-like Gene Is Required for Stem Cell Maintenance in Planarians,” Dev. Cell, vol. 11, no. 2, pp. 159–169, 2006 doi: 10.1016/j.devcel.2006.06.004 16890156 10.1016/j.devcel.2006.06.004

[48] Demircan T. and Berezikov E., “The Hippo pathway regulates stem cells during homeostasis and regeneration of the flatworm Macrostomum lignano.,” Stem Cells Dev., vol. 22, no. 15, pp. 2174–85, 2013 doi: 10.1089/scd.2013.0006 23495768 10.1089/scd.2013.0006

[49] Halder G. and Johnson R. L., “Hippo signaling: growth control and beyond.,” Development, vol. 138, no. 1, pp. 9–22, 2011 doi: 10.1242/dev.045500 21138973 10.1242/dev.045500 PMC2998162

[50] Gao T., Zhou D., Yang C., Singh T., Penzo A.-, Maddipati R., Tzatsos A., and Bardeesy N., “Hippo Signaling Regulates Differentiation and Maintenance in the Exocrine Pancreas,” Gastroenterology, vol. 144, no. 7, pp. 1543–1553, 2014. 10.1053/j.gastro.2013.02.037 PMC3665616 23454691

[51] Oh H. J., Kim M. J., Song S. J., Kim T., Lee D., Kwon S. H., Choi E. J., and Lim D. S., “MST1 Limits the Kinase Activity of Aurora B to Promote Stable Kinetochore-Microtubule Attachment,” Curr. Biol., vol. 20, no. 5, pp. 416–422, 2010 doi: 10.1016/j.cub.2009.12.054 20171103 10.1016/j.cub.2009.12.054

[52] Yabuta N., Mukai S., Okamoto A., Okuzaki D., Suzuki H., Torigata K., Yoshida K., Okada N., Miura D., Ito A., Ikawa M., Okabe M., and Nojima H., “N-terminal truncation of Lats1 causes abnormal cell growth control and chromosomal instability.,” J. Cell Sci., vol. 126, no. Pt 2, pp. 508–520, 2013 doi: 10.1242/jcs.113431 23230145 10.1242/jcs.113431

[53] Ganem N. J., Cornils H., Chiu S. Y., O’Rourke K. P., Arnaud J., Yimlamai D., Théry M., Camargo F. D., and Pellman D., “Cytokinesis failure triggers hippo tumor suppressor pathway activation,” Cell, vol. 158, no. 4, pp. 833–848, 2014 doi: 10.1016/j.cell.2014.06.029 25126788 10.1016/j.cell.2014.06.029 PMC4136486

[54] Orii H., Sakurai T., and Watanabe K., “Distribution of the stem cells (neoblasts) in the planarian Dugesia japonica,” Dev. Genes Evol., vol. 215, no. 3, pp. 143–157, 2005 doi: 10.1007/s00427-004-0460-y 15657737 10.1007/s00427-004-0460-y

[55] Dong J., Feldmann G., Huang J., Wu S., Zhang N., Comerford S. A., Gayyed M. F., Anders R. A., Maitra A., and Pan D., “Elucidation of a Universal Size-Control Mechanism in Drosophila and Mammals,” Cell, vol. 130, no. 6, pp. 1120–1133, 2007 doi: 10.1016/j.cell.2007.07.019 17889654 10.1016/j.cell.2007.07.019 PMC2666353

[56] Skibinski A., Breindel J. L., Prat A., Galvín P., Smith E., Rolfs A., Gupta P. B., LaBaer J., and Kuperwasser C., “The Hippo Transducer TAZ Interacts with the SWI/SNF Complex to Regulate Breast Epithelial Lineage Commitment,” Cell Rep., vol. 6, no. 6, pp. 1059–1072, 2014 doi: 10.1016/j.celrep.2014.02.038 24613358 10.1016/j.celrep.2014.02.038 PMC4011189

[57] Cordenonsi M., Zanconato F., Azzolin L., Forcato M., Rosato A., Frasson C., Inui M., Montagner M., Parenti A. R., Poletti A., Daidone M. G., Dupont S., Basso G., Bicciato S., and Piccolo S., “The Hippo transducer TAZ confers cancer stem cell-related traits on breast cancer cells,” Cell, vol. 147, no. 4, pp. 759–772, 2011 doi: 10.1016/j.cell.2011.09.048 22078877 10.1016/j.cell.2011.09.048

[58] Zhivotovsky B. and Kroemer G., “Apoptosis and genomic instability.,” Nat. Rev. Mol. Cell Biol., vol. 5, no. 9, pp. 752–762, 2004 doi: 10.1038/nrm1443 15340382 10.1038/nrm1443

[59] Almuedo-Castillo M., Crespo X., Seebeck F., Bartscherer K., Saló E., and Adell T., “JNK Controls the Onset of Mitosis in Planarian Stem Cells and Triggers Apoptotic Cell Death Required for Regeneration and Remodeling,” PLoS Genet., vol. 10, no. 6, pp. 1–15, 2014. 10.1371/journal.pgen.1004400 PMC4055413 24922054

[60] Witchley J. N., Mayer M., Wagner D. E., Owen J. H., and Reddien P. W., “Muscle Cells Provide Instructions for Planarian Regeneration,” Cell Rep., vol. 4, no. 4, pp. 633–641, 2013 doi: 10.1016/j.celrep.2013.07.022 23954785 10.1016/j.celrep.2013.07.022 PMC4101538

[61] Gotzmann J., Mikula M., Eger A., Schulte-Hermann R., Foisner R., Beug H., and Mikulits W., “Molecular aspects of epithelial cell plasticity: implications for local tumor invasion and metastasis.,” Mutat. Res., vol. 566, no. 1, pp. 9–20, 2004 14706509 10.1016/s1383-5742(03)00033-4

[62] Yanger K., Zong Y., Maggs L. R., Shapira S. N., Maddipati R., Aiello N. M., Thung S. N., Wells R. G., Greenbaum L. E., and Stanger B. Z., “Robust cellular reprogramming occurs spontaneously during liver regeneration,” Genes Dev., vol. 27, no. 7, pp. 719–724, 2013 doi: 10.1101/gad.207803.112 23520387 10.1101/gad.207803.112 PMC3639413

[63] Kikuchi K., “Dedifferentiation, Transdifferentiation, and Proliferation: Mechanisms Underlying Cardiac Muscle Regeneration in Zebrafish.,” Curr. Pathobiol. Rep., vol. 3, no. 1, pp. 81–88, 2015 doi: 10.1007/s40139-015-0063-5 25722956 10.1007/s40139-015-0063-5 PMC4333235

[64] Cebrià F. and a Newmark P., “Planarian homologs of netrin and netrin receptor are required for proper regeneration of the central nervous system and the maintenance of nervous system architecture.,” Development, vol. 132, no. 16, pp. 3691–3703, 2005 doi: 10.1242/dev.01941 16033796 10.1242/dev.01941

[65] Sanchez Alvarado A. and Newmark P. A., “Double-stranded RNA specifically disrupts gene expression during planarian regeneration,” Dev. Biol., vol. 96, pp. 5049–5054, 1999. 10.1073/pnas.96.9.5049 PMC21814 10220416

[66] Currie K. W., Brown D. D. R., Zhu S., Xu C., Voisin V., Bader G. D., and Pearson B. J., “HOX gene complement and expression in the planarian Schmidtea mediterranea.,” Evodevo, vol. 7, no. 7, pp. 1–23, 2016. 27034770 10.1186/s13227-016-0044-8 PMC4815179

[67] King R. S. and Newmark P. A., “In situ hybridization protocol for enhanced detection of gene expression in the planarian Schmidtea mediterranea.,” BMC Dev. Biol., vol. 13, no. 8, pp. 1–32, 2013. 23497040 10.1186/1471-213X-13-8 PMC3610298

[68] Ross K. G., Omuro K. C., Taylor M. R., Munday R. K., Hubert A., King R. S., and Zayas R. M., “Novel monoclonal antibodies to study tissue regeneration in planarians.,” BMC Dev. Biol., vol. 15, p. 2, 2015 doi: 10.1186/s12861-014-0050-9 25604901 10.1186/s12861-014-0050-9 PMC4307677

[69] Barberán S., Fraguas S., and Cebrià F., “The EGFR signaling pathway controls gut progenitor differentiation during planarian regeneration and homeostasis,” Development, vol. 143, no. 12, pp. 2089–2102, 2016 doi: 10.1242/dev.131995 27122174 10.1242/dev.131995

[70] Pellettieri J. and Sánchez Alvarado A., “Cell turnover and adult tissue homeostasis: from humans to planarians.,” Annu. Rev. Genet., vol. 41, pp. 83–105, 2007 doi: 10.1146/annurev.genet.41.110306.130244 18076325 10.1146/annurev.genet.41.110306.130244

[71] González-Estévez C., Felix D. A., Aboobaker A. A., and Saló E., “Erratum: Gtdap-1 and the role of autophagy during planarian regeneration and starvation (Autophagy),” Proc. Natl. Acad. Sci. U. S. A., vol. 3, no. 6, pp. 640–642, 2007. 10.4161/auto.4934 17786038

[72] Moritz S., Stöckle F., Ortmeier C., Schmitz H., Rodríguez-Esteban G., Key G., and Gentile L., “Heterogeneity of planarian stem cells in the S/G2/M phase,” Int. J. Dev. Biol., vol. 56, no. 1–3, pp. 117–125, 2012 doi: 10.1387/ijdb.113440sm 22450999 10.1387/ijdb.113440sm

[73] Sureda-Gómez M., Martín-Durán J. M., and Adell T., “Localization of planarian βCATENIN-1 reveals multiple roles during anterior-posterior regeneration and organogenesis.,” Development, vol. 143, no. 22, pp. 4149–4160, 2016 doi: 10.1242/dev.135152 27737903 10.1242/dev.135152

[74] Bray N. L., Pimentel H., Melsted P., and Pachter L., “Near-optimal probabilistic RNA-seq quantification,” Nat. Biotechnol., vol. 34, no. 5, pp. 525–527, 2016 doi: 10.1038/nbt.3519 27043002 10.1038/nbt.3519

[75] Brandl H., Moon H. K., Vila-Farré M., Liu S. Y., Henry I., and Rink J. C., “PlanMine—A mineable resource of planarian biology and biodiversity,” Nucleic Acids Res., vol. 44, no. D1, pp. 764–773, 2016. 10.1093/nar/gkv1148 PMC4702831 26578570

[76] Pimentel H. J., Bray N., Puente S., Melsted P., and Pachter L., “Differential analysis of RNA-Seq incorporating quantification uncertainty,” Nat. Methods, vol. 14, pp. 687–690, 2017 doi: 10.1038/nmeth.4324 28581496 10.1038/nmeth.4324

[77] Love M. I., Huber W., and Anders S., “Moderated estimation of fold change and dispersion for RNA-seq data with DESeq2.,” Genome Biol., vol. 15, no. 12, p. 550, 2014 doi: 10.1186/s13059-014-0550-8 25516281 10.1186/s13059-014-0550-8 PMC4302049

[78] Barrett T., Wilhite S. E., Ledoux P., Evangelista C., Kim I. F., Tomashevsky M., Marshall K. A., Phillippy K. H., Sherman P. M., Holko M., Yefanov A., Lee H., Zhang N., Robertson C. L., Serova N., Davis S., and Soboleva A., “NCBI GEO: Archive for functional genomics data sets—Update,” Nucleic Acids Res., vol. 41, no. D1, pp. 991–995, 2013. 10.1093/nar/gks1193 PMC3531084 23193258

[79] Rodríguez-Esteban G, de Sousa N, Adell T, Saló E. Smed-hippo(RNAi) RNA-Seq [dataset]. [cited 2018 Jan 18]. Gene Expression Omnibus. Available from: https://www.ncbi.nlm.nih.gov/geo/query/acc.cgi?acc=GSE95130

