# Supplementary figures and images for: Hippo signaling controls cell cycle and restricts cell plasticity in planarians
Source: PLoS Biol. 2018 Jan 22;16(1):e2002399. doi: 10.1371/journal.pbio.2002399 (PMC5794332; doi:10.1371/journal.pbio.2002399)

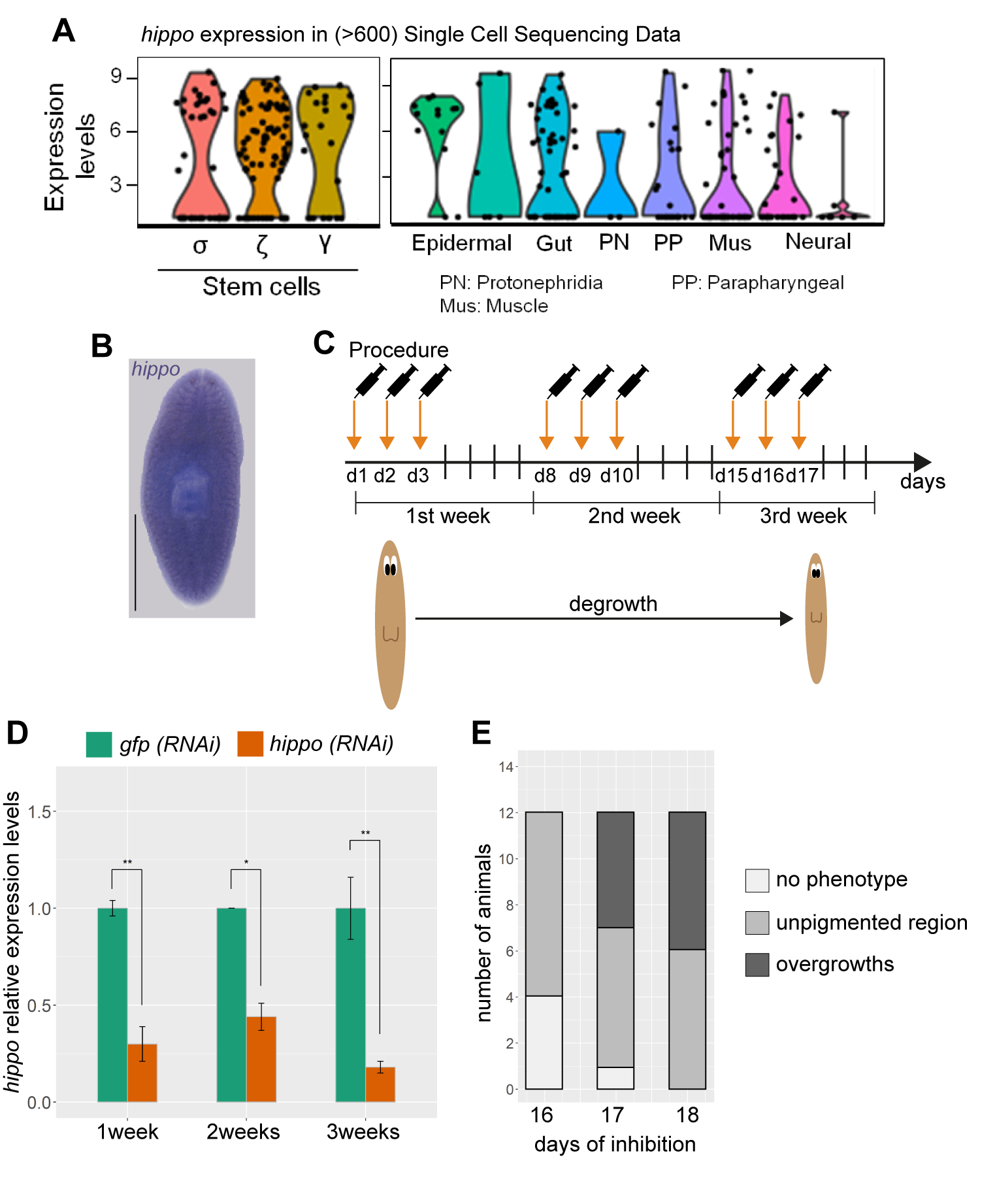

Supplement: S1 Fig — (A) hippo expression levels in different cell types according to a single-cell RNAseq analysis (74). (B) In situ hybridization for hippo reveals a ubiquitous expression pattern. (C) Cartoon illustrating the experimental design used for hippo RNAi during planarian homeostasis. Animals were starved for 1 week before the experiment and were then injected on 3 consecutive days each week for 3 weeks. Starvation was maintained throughout. (D) Relative expression levels of hippo after hippo RNAi, as measured by qRT-PCR. Values represent the means of 3 biological replicates. Error bars represent standard deviation. Data were analyzed by Student t test. *p < 0.05; **p < 0.01. (E) Graph showing the evolution of hippo (RNAi) phenotypes after 16, 17, and 18 days of hippo inhibition. Data used in the generation of this figure can be found in S1 Data. RNAi, RNA interference; RNAseq, RNA sequencing analysis. (TIF) [file pbio.2002399.s001.tif]

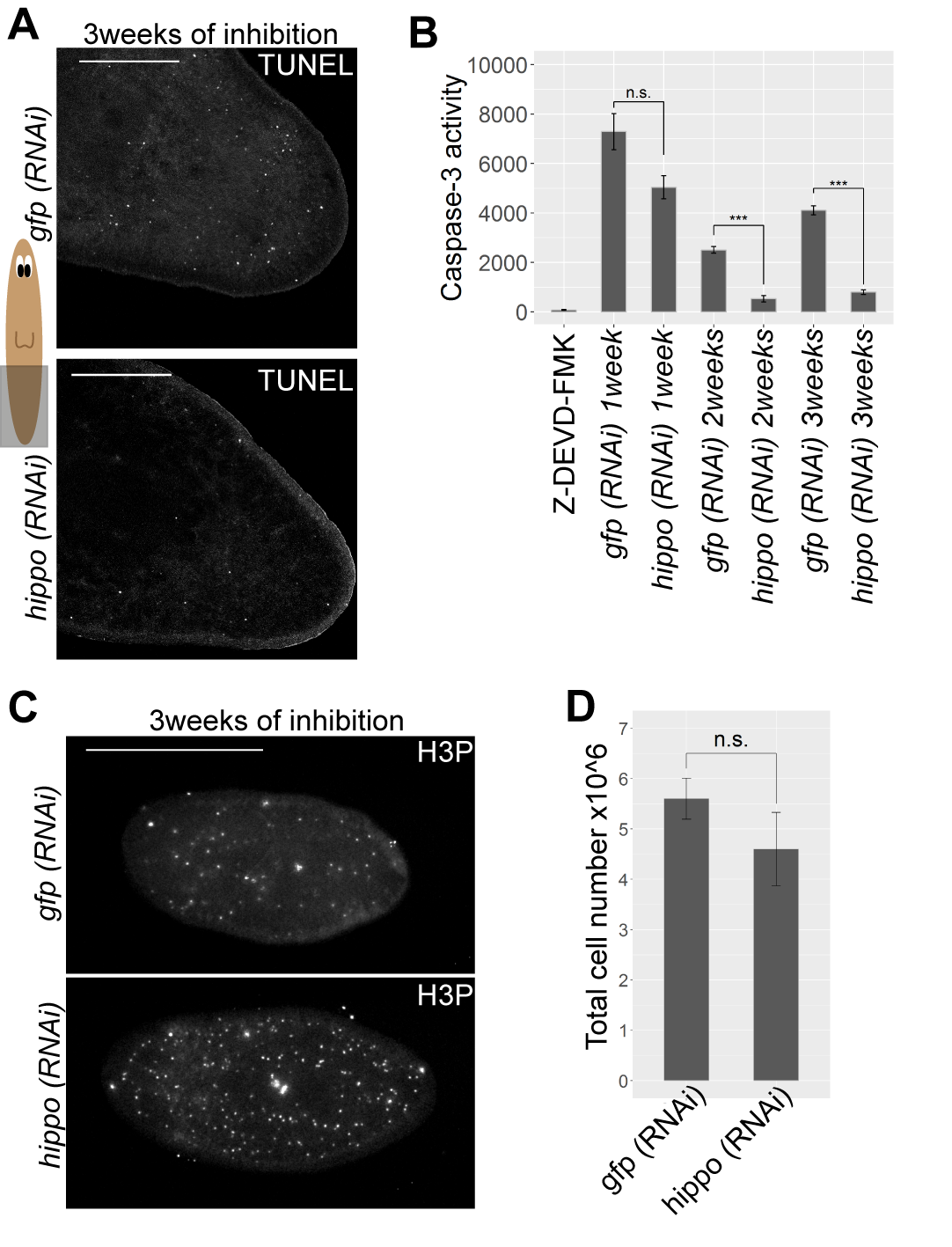

Supplement: S2 Fig — (A) Whole-mount TUNEL showing apoptotic cell death in planarians subjected to hippo RNAi for 3 weeks (n ≥ 5). Images correspond to confocal Z-projections. (B) Quantification of caspase-3 activity after 1, 2, and 3 weeks of hippo inhibition. Results are presented as units of caspase-3 activity per μg of protein. Bars correspond to the mean of 3 biological replicates. Error bars represent standard deviation. (C) Immunostaining with anti-H3P antibody in planarians subjected to hippo RNAi for 3 weeks (n ≥ 10). (D) Graph showing the total cell number in planarians subjected to hippo RNAi for 3 weeks, as determined using a Neubauer chamber. Bars correspond to the mean of 3 biological replicates. Error bars represent standard deviation. Data were analyzed by Student t test. **p < 0.01; ***p < 0.001. Data used in the generation of this figure can be found in S1 Data. Scale bars: 250 μm (A); 1 mm (B). n.s., not significant; RNAi, RNA interference. (TIF) [file pbio.2002399.s002.tif]

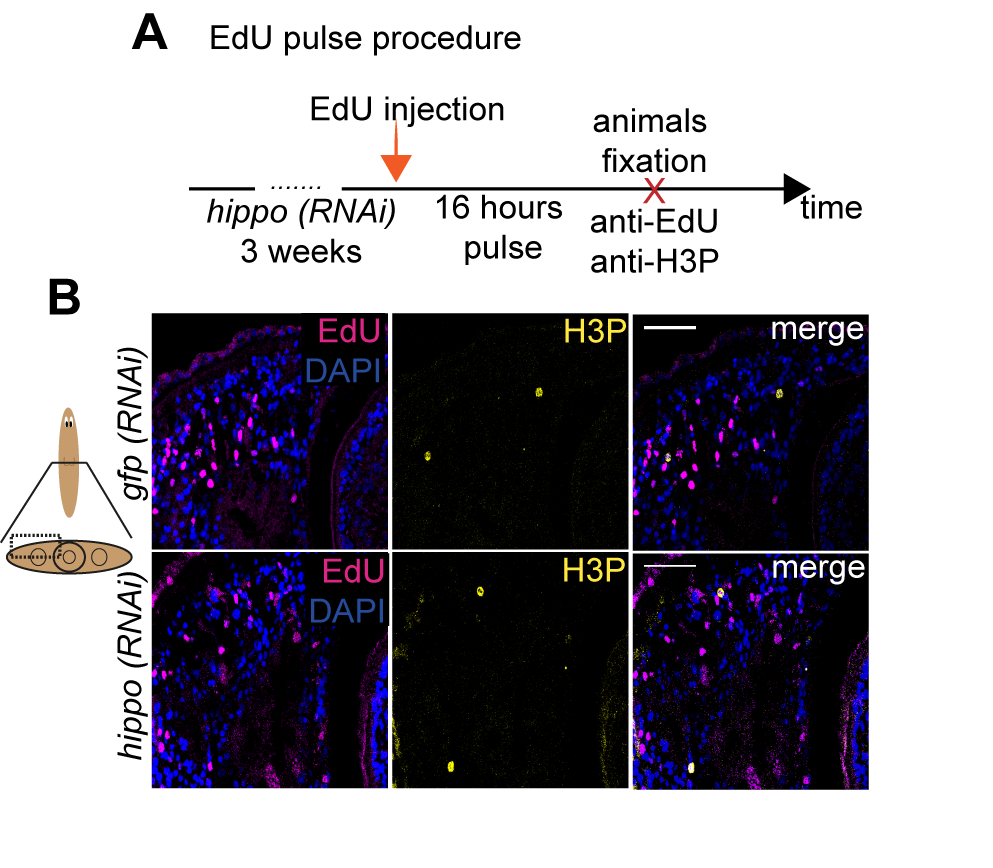

Supplement: S3 Fig — (A) Cartoon illustrating the EdU pulse procedure. Animals were starved for 1 week, injected with hippo dsRNA for 3 weeks, and then injected with EdU and fixed 16 h later. (B) EdU labeling in transverse sections combined with immunostaining with anti-H3P antibody in the pharynx region in controls and in planarians subjected to hippo RNAi for 3 weeks. Scale bars: 50 μm. dsRNA, double-stranded RNA; EdU, 5-ethynyl-2′-deoxyuridine; H3P, phospho-histone-H3-Ser10; RNAi, RNA interference. (TIF) [file pbio.2002399.s003.tif]

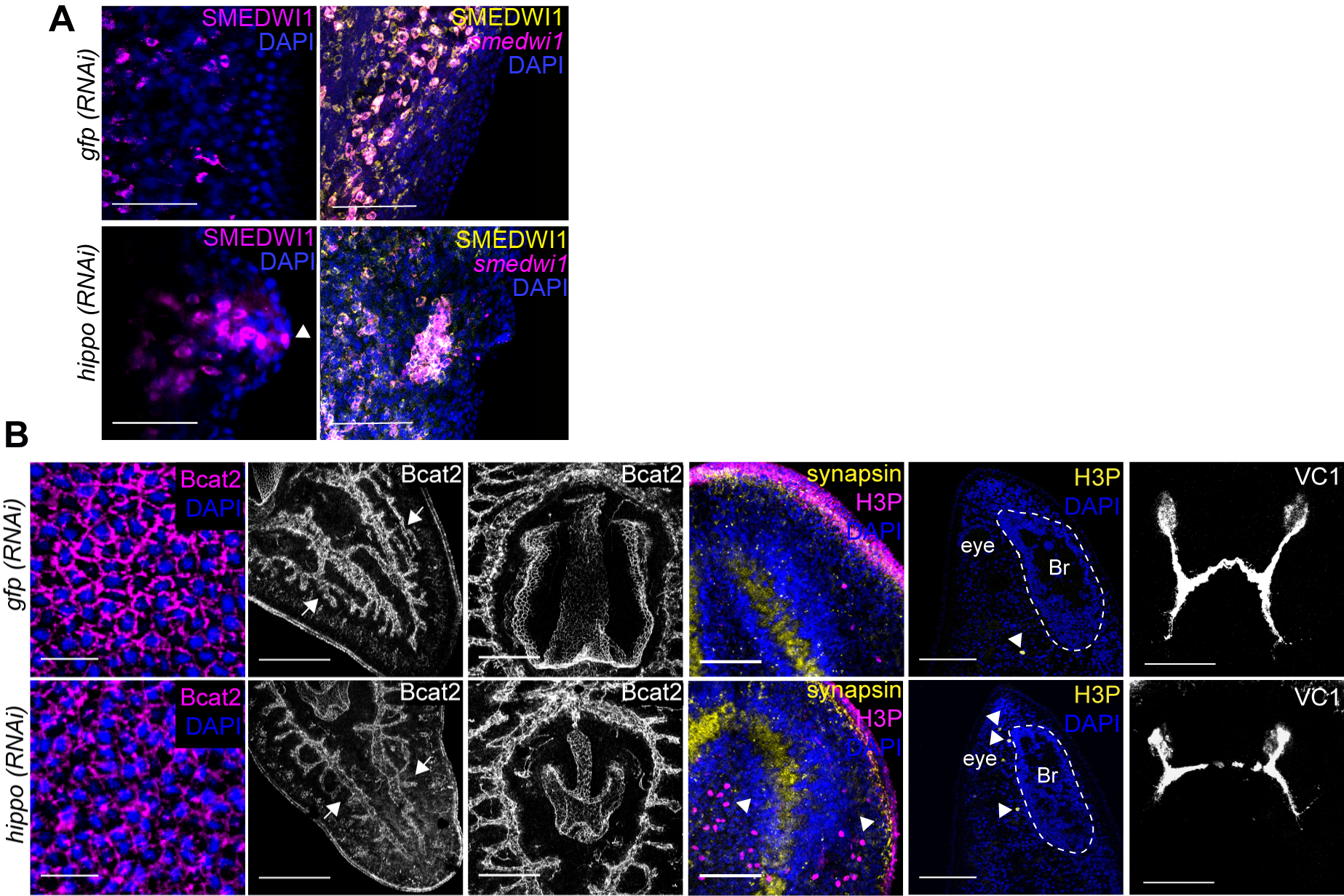

Supplement: S4 Fig — (A) Analysis of overgrowths. FISH combined with immunostaining showing the localization of smedwi-1 mRNA and SMEDWI-1 protein. Colocalization of both signals appears to be concentrated in the overgrowths, indicating that they consist of undifferentiated cells. Arrowhead indicates an epidermal cell of an overgrowth stained with SMEDWI-1. (B) Analysis of unpigmented regions. Immunostaining using different markers. From left to right: staining of the epithelia with anti-anti-Bcat2 antibody; digestive system labeled with anti-Bcat2 antibody (white arrows indicate gut branches); pharynx labeled with anti-Bcat2 antibody; head region stained with anti-synapsin, anti-H3P, and anti-Bcat2 antibodies (arrowheads indicate mitotic cells); sagittal section showing a head region stained with anti-H3P (arrowheads indicate mitotic cells; discontinuous line delimits the brain); visual system stained with anti-arrestin (VC-1). Blue corresponds to nuclei stained with DAPI. All experiments were performed in planarians subjected to hippo RNAi for 3 weeks. All images correspond to confocal Z-projections. Scale bars: 100 μm; 200 μm (A); 100 μm; 250 μm; 150 μm; 250 μm; 150 μm; 100 μm (B). Bcat2, β-catenin-2; Br, brain; FISH, fluorescent in situ hybridization; H3P, phospho-histone-H3-Ser10; RNAi, RNA interference. (TIF) [file pbio.2002399.s004.tif]

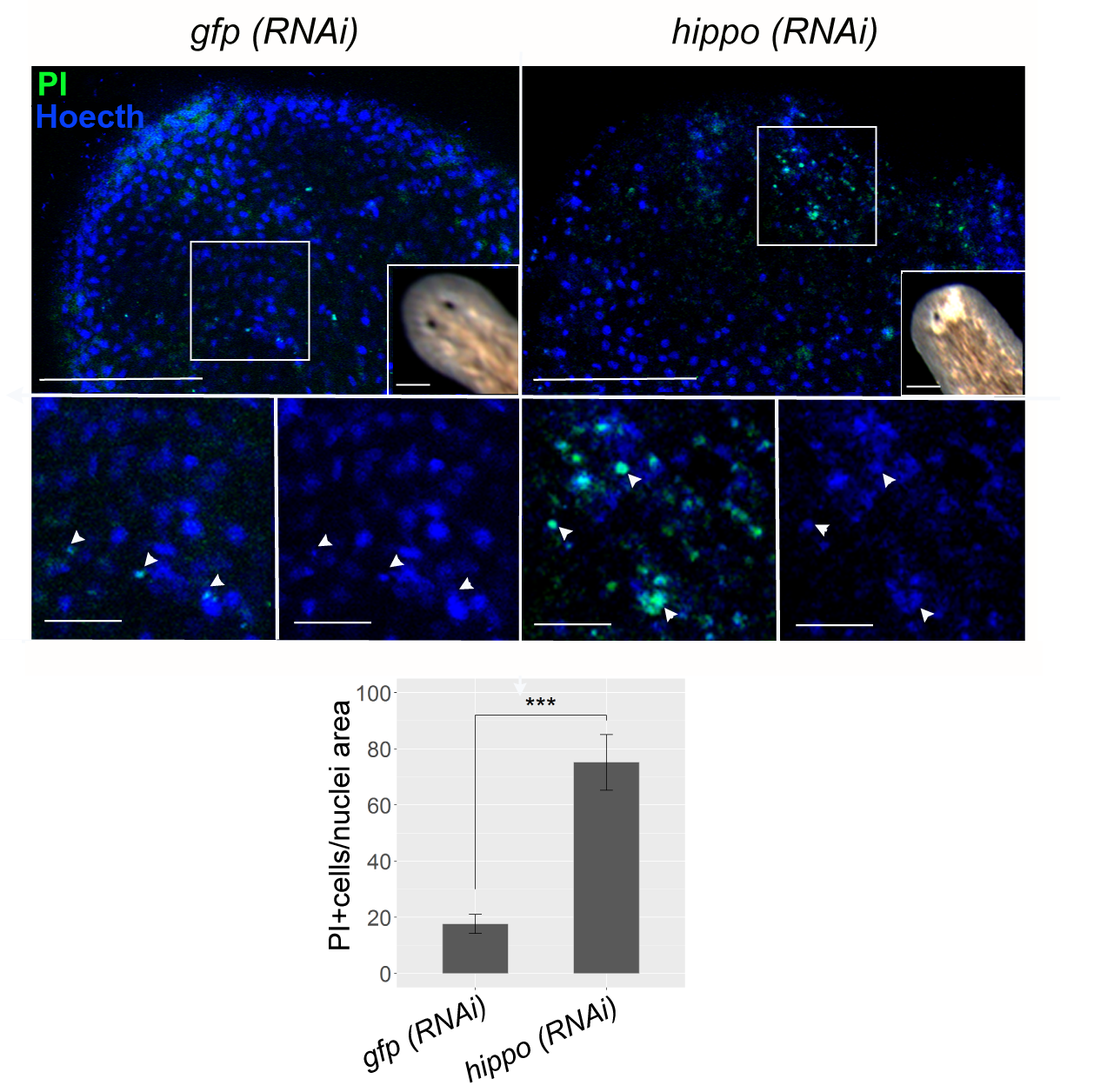

Supplement: S5 Fig — Staining of dead cells using PI in live control and hippo (RNAi) animals. Nuclei are stained with Hoechst. Magnifications of the indicated area are shown below. Arrowhead indicates some cells positive for PI. A stereomicroscopic view of live control and hippo (RNAi) animals used in the experiment is shown. Quantification of the PI+ cells per nuclei area in the head region is shown. Data were analyzed by Student t test (n = 4). ***p < 0.001. Data used in the generation of this figure can be found in S1 Data. Scale bars: 100 μm (top images); 25 μm (bottom images). PI, propidium iodide; RNAi, RNA interference. (TIF) [file pbio.2002399.s005.tif]

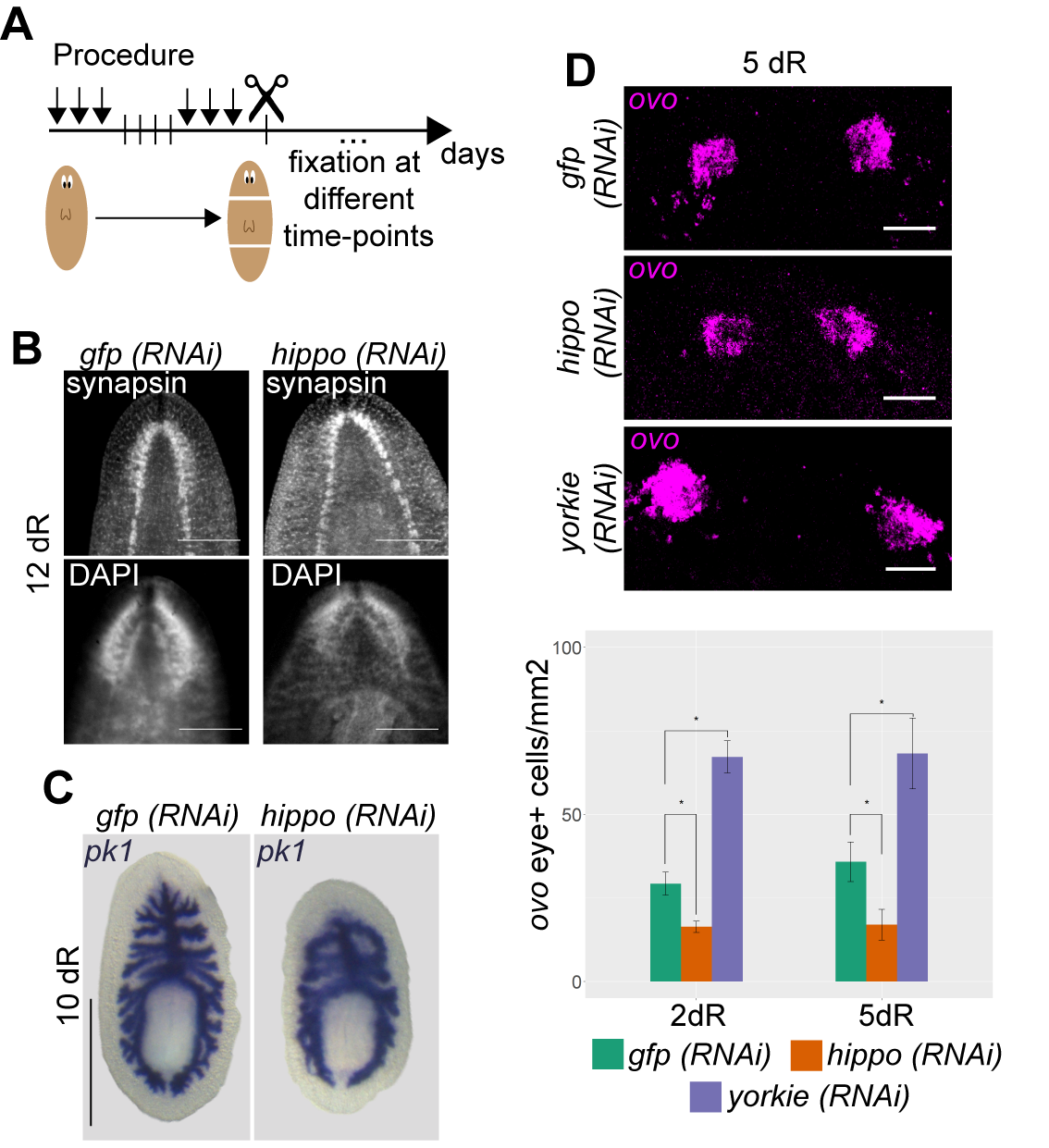

Supplement: S6 Fig — (A) Cartoon illustrating the RNAi procedure in regenerating conditions. Animals were starved for 1 week before the experiment and then injected on 3 consecutive days. The following week, animals were injected again on 3 consecutive days, cut the next day, and fixed at different time points. (B) Anti-synapsin immunostaining of the brain region of control and hippo (RNAi) animals. Nuclei are stained with DAPI. Images correspond to planarians after 12 dR. (C) In situ hybridization with pk1 (digestive system) in hippo (RNAi) and control animals. Images correspond to planarians after 10 dR. (D) Fluorescent in situ hybridization for ovo to label eyes in hippo (RNAi), yorkie (RNAi), and control animals. Images correspond to planarians after 5 dR. The corresponding quantification is shown. Data were analyzed by Student t test. *p < 0.05. Data used in the generation of this figure can be found in S1 Data. Scale bars: 200 μm (B); 0.5 mm (C); 50 μm (D). dR, days of regeneration; pk1, pantothenate kinase 1; RNAi, RNA interference. (TIF) [file pbio.2002399.s006.tif]

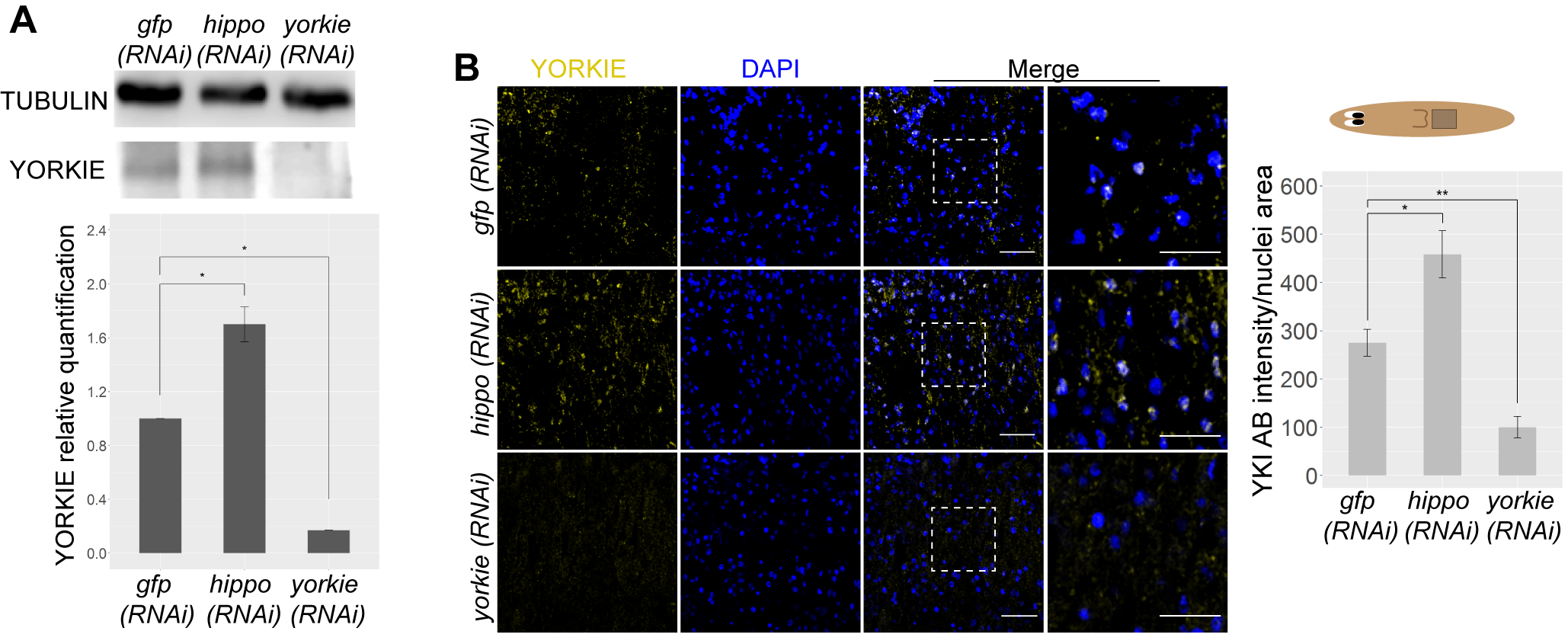

Supplement: S7 Fig — (A) Western blot of protein extracts from hippo (RNAi), yorkie (RNAi), and control planarians immunoblotted with anti-Yorkie and anti-α-Tubulin antibodies. Extracts correspond to intact animals after 3 weeks (hippo (RNAi)) or 1 week (yorkie (RNAi)) of RNAi. The corresponding quantification of the Yorkie versus Tubulin signal is shown. Bars represent the mean of 3 biological replicates. (B) Immunostaining with anti-Yorkie in longitudinal sections of hippo (RNAi), yorkie (RNAi), and control planarians. hippo was inhibited for 3 consecutive weeks and yorkie for 1 week. Nuclei were stained with DAPI. Magnifications of the indicated region are included. The corresponding quantification of the nuclear signal in the post-pharyngeal region is shown. Yorkie nuclear signal intensity was measured as RID and then normalized to the corresponding nuclear area (see Materials and methods). Error bars represent standard deviation (n ≥ 3). Data were analyzed by Student t test. *p < 0.05; **p < 0.01. Data used in the generation of this figure can be found in S1 Data. Scale bars: 50 μm (B). RID, raw integrated density; RNAi, RNA interference. (TIF) [file pbio.2002399.s007.tif]

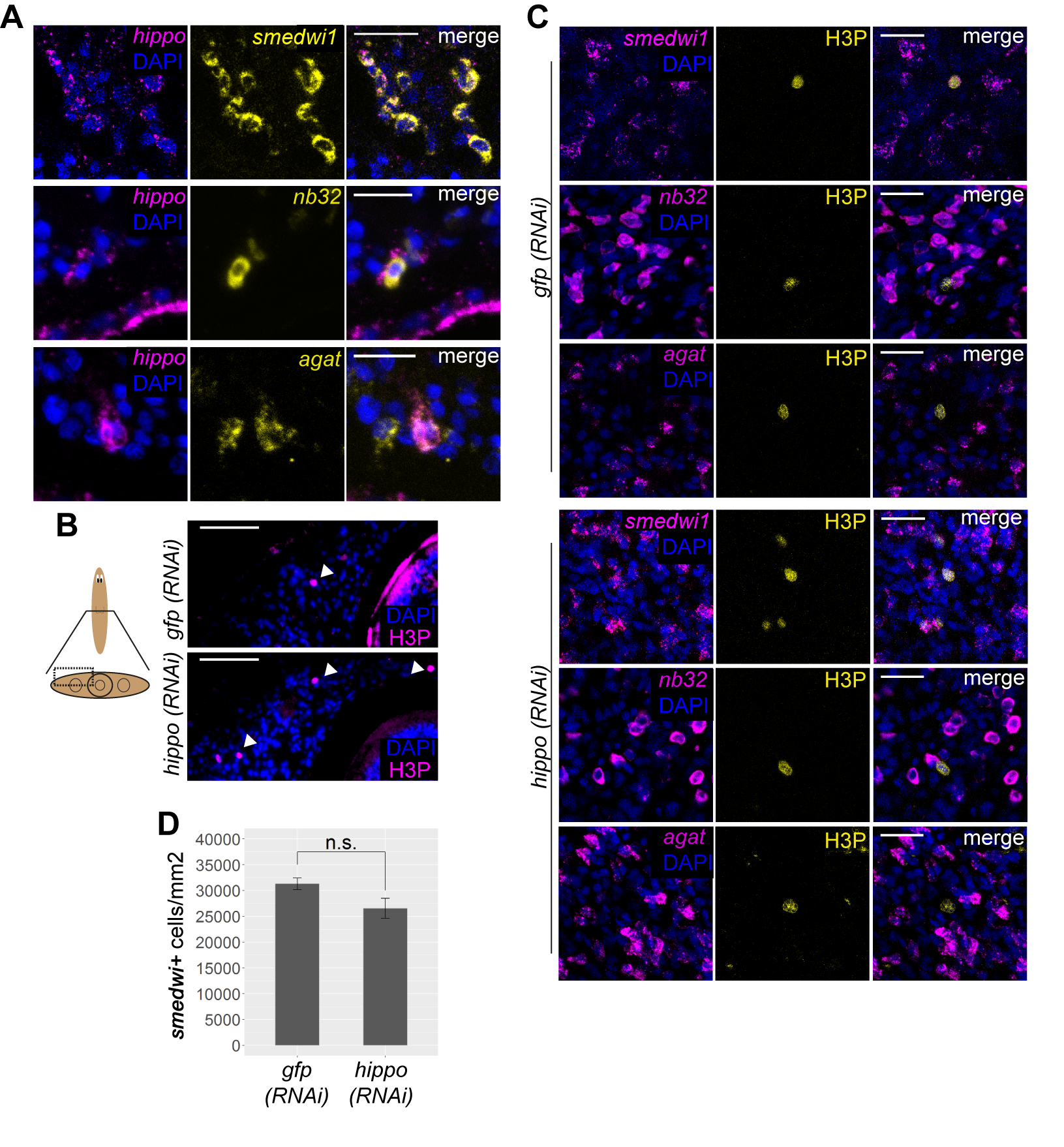

Supplement: S8 Fig — (A) FISH showing colocalization of hippo with smedwi-1, nb32, and agat. (B) Immunostaining using anti-H3P antibody in transverse sections. Nuclei are stained with DAPI. White arrows indicate H3P+ cells. (C) FISH with smedwi-1, nb32, and agat combined with immunostaining with anti-H3P. Nuclei are stained with DAPI. (D) Graph showing the quantification of smedwi-1+ cells in control and hippo (RNAi) animals. smedwi-1+ cells were quantified in the posterior half and normalized with respect to the area quantified. Error bars represent the standard deviation (n ≥ 7). Data were analyzed by Student t test. Data used in the generation of this figure can be found in S1 Data. Scale bars: 15 μm (A); 100 μm (B); 25 μm (C). FISH, fluorescent in situ hybridization; H3P, phospho-histone-H3-Ser10; n.s., not significant; RNAi, RNA interference. (TIF) [file pbio.2002399.s008.tif]

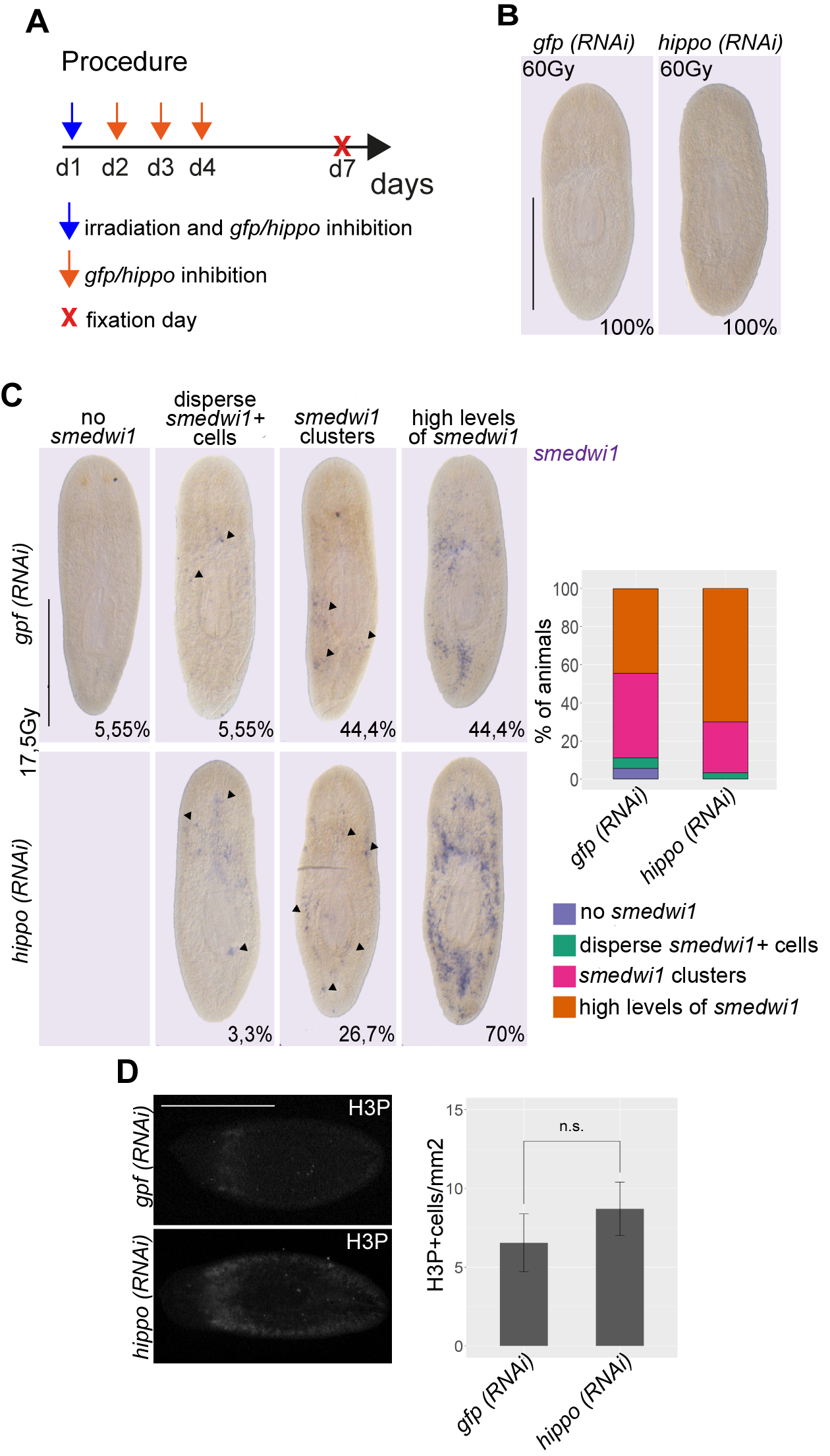

Supplement: S9 Fig — (A) Cartoon illustrating the irradiation and dsRNA injection procedures. Animals were starved for 1 week, exposed to different doses of radiation (60 Gy and 17.5 Gy), and injected with hippo or gfp dsRNA for 4 consecutive days. Animals were fixed 7 days after the beginning of the experiment. (B) In situ hybridization for smedwi-1 probe in animals irradiated with 60 Gy. No signal is detected (n ≥ 18). (C) In situ hybridization for smedwi-1 in gfp and hippo (RNAi) animals irradiated with 17.5 Gy. Black arrows indicate smedwi-1+ cells/clusters. The corresponding quantification of smedwi-1 expression, distribution, and classification into 4 categories is shown (n ≥ 18). Data were analyzed by chi-squared test, applying a Bonferroni correction (*p < 0.05). (D) Anti-H3P immunostaining in gfp and hippo (RNAi) animals irradiated with 17.5 Gy. Corresponding quantification is shown. H3P+ cells in the whole animal were quantified and normalized to the total area of each animal. Error bars represent standard deviation (n ≥ 13). Data were analyzed by Student t test. Data used in the generation of this figure can be found in S1 Data. Scale bars: 1 mm. dsRNA, double-stranded RNA; gfp, green fluorescent protein; H3P, phospho-histone-H3-Ser10; n.s., not significant; RNAi, RNA interference. (TIF) [file pbio.2002399.s009.tif]

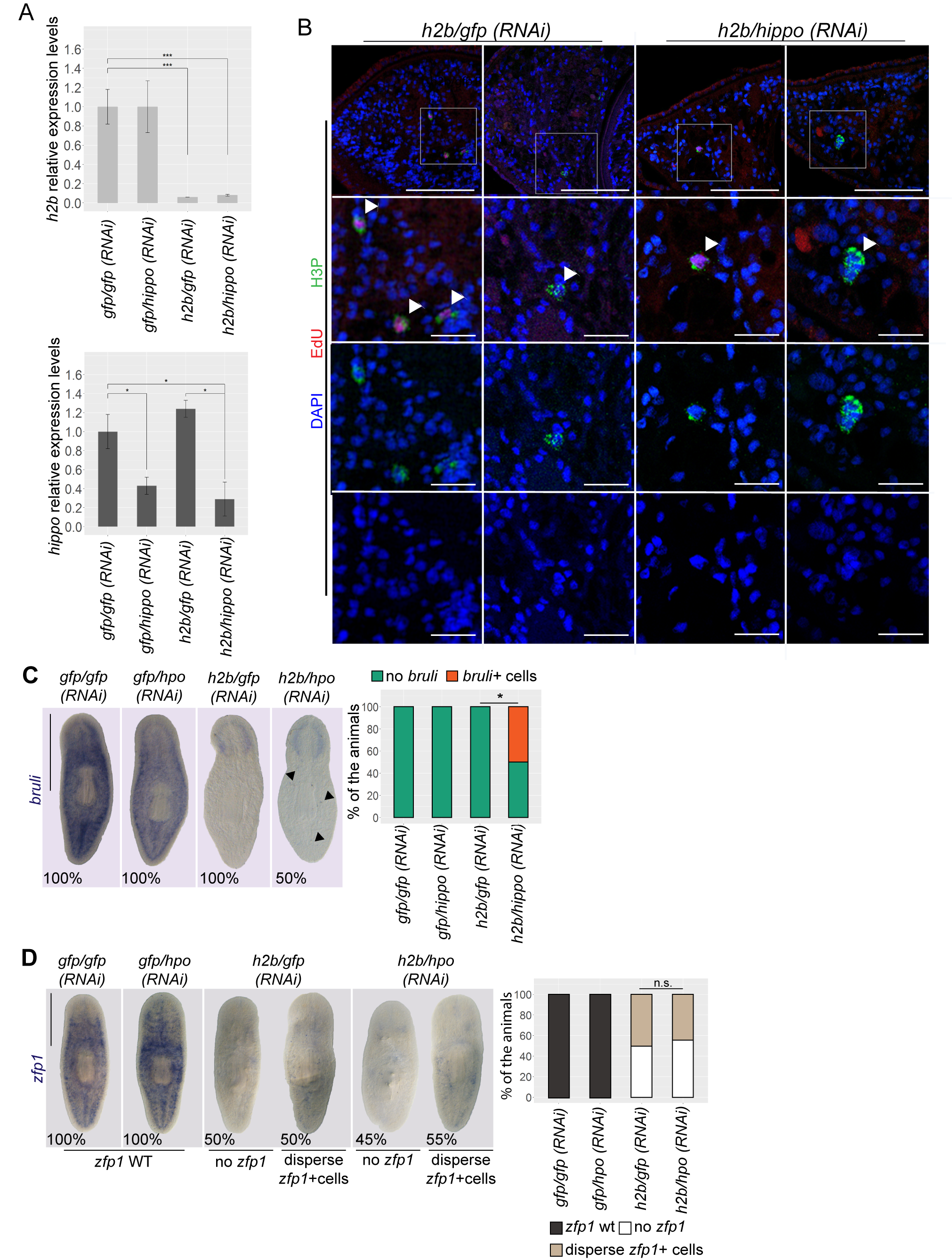

Supplement: S10 Fig — (A) Relative expression of h2b and hippo, as determined by qRT-PCR, in planarians 4 days postinjection. Values represent the means of 3 biological replicates. Error bars represent standard deviation. Data were analyzed by Student t test. *p < 0.05; ***p < 0.001. (B) EdU labeling in transverse paraffin sections combined with immunostaining with anti-H3P antibody in h2b/gfp (RNAi) and h2b/hippo (RNAi) animals. Arrowhead indicates smedwi-1+ cells, some of which are EdU+. Fifteen sections corresponding to n ≥ 8 animals were analyzed. (C) In situ hybridization for bruli in h2b/hippo (RNAi) animals and corresponding controls. Black arrows indicate bruli+ cells. Quantification of bruli expression and classification into 2 categories (n ≥ 6). Data were analyzed by chi-squared test, applying a Bonferroni correction; *p < 0.05. (D) In situ hybridization for zfp1 in the 4 RNAi conditions, corresponding quantification of zfp1 expression, and classification into 4 categories (n ≥ 10). Data were analyzed by chi-squared test, applying a Bonferroni correction. Data used in the generation of this figure can be found in S1 Data. Scale bars: 15 μm (B); 1 mm (C and D). EdU, 5-ethynyl-2′-deoxyuridine; h2b, histone 2b; H3P, phospho-histone-H3-Ser10; RNAi, RNA interference. (TIF) [file pbio.2002399.s010.tif]
